# A dual-level Bayesian network meta-analysis of first-line immunotherapy in advanced hepatocellular carcinoma: strategy-level and regimen-level evidence

**DOI:** 10.3389/fimmu.2026.1907830

**Published:** 2026-08-05

**Authors:** Qiangqiang Zhang, Yanyan Hu, Yuli Ge, Rui Wang

**Affiliations:** 1 Department of Medical Oncology, Jinling Hospital, Affiliated Hospital of Medical School, Nanjing University, Nanjing, China; 2 Department of Medical Oncology, Jinling Hospital, Nanjing Medical University, Nanjing, China; 3 Department of Medical Oncology, Jinling Clinical Medical College, Nanjing University of Chinese Medicine, Nanjing, China

**Keywords:** Bayesian network meta-analysis, first-line treatment, hepatocellular carcinoma, immunotherapy combinations, ranking

## Abstract

**Background:**

Phase III trials of first-line immunotherapy-based combinations for advanced hepatocellular carcinoma (HCC) have yielded heterogeneous results, and head-to-head comparisons remain scarce. We evaluated mechanism-based strategy classes and individual regimens using a dual-level Bayesian network meta-analysis to provide a structured interpretation of the current evidence.

**Methods:**

We systematically searched the literature up to December 28, 2025, and identified 15 phase III randomised controlled trials. A dual-level Bayesian network meta-analysis was performed using separate models at the strategy level (PD-(L)1 plus VEGF monoclonal antibody (VEGF(Ab)), PD-(L)1 plus tyrosine kinase inhibitor (TKI), PD-(L)1 plus CTLA-4, and PD-(L)1 monotherapy) and the regimen level. Outcomes included overall survival (OS), progression-free survival (PFS), objective response rate (ORR), and grade ≥3 treatment-related adverse events (TRAEs).

**Results:**

Fifteen trials comprising 9,792 patients were included. At the strategy level, PD-(L)1 plus VEGF(Ab) showed the most favourable OS effect versus TKI monotherapy and the highest efficacy-oriented ranking, followed by PD-(L)1 plus CTLA-4 and PD-(L)1 plus TKI. PD-(L)1 plus TKI also improved PFS but was associated with a higher toxicity and discontinuation burden, whereas PD-(L)1 monotherapy showed the most favourable tolerability profile. At the regimen level, atezolizumab plus bevacizumab, sintilimab plus IBI305, camrelizumab plus rivoceranib, and STRIDE showed OS benefit versus sorafenib.

**Conclusions:**

In this evidence network, PD-(L)1 plus VEGF(Ab) showed the most efficacy-oriented profile and was supported by relatively consistent regimen-level estimates. However, the clinical interpretation of strategy-level findings differed across classes: PD-(L)1 plus TKI required closer regimen-level assessment because of greater within-class variability and a less favourable toxicity profile, whereas PD-(L)1 plus CTLA-4 and PD-(L)1 monotherapy showed distinct benefit-risk patterns. This dual-level framework complements regimen-centric evidence by distinguishing strategy-level patterns from regimen-level findings and by supporting a structured interpretation of efficacy-toxicity trade-offs in first-line ICI-based therapy for advanced HCC.

**Systematic Review Registration:**

https://www.crd.york.ac.uk/PROSPERO/view/CRD420251273309, identifier CRD420251273309.

## 1 Introduction

Hepatocellular carcinoma (HCC), the most common form of primary liver cancer, remains a major global health burden and caused approximately 757,000 deaths in 2022 (1). Prognosis is still poor, mainly because many patients are diagnosed at an advanced stage. This is reflected by a 5-year survival rate of approximately 3.5% among patients with distant-stage disease (2). For more than a decade, first-line systemic therapy was largely based on tyrosine kinase inhibitors (TKIs), such as sorafenib and lenvatinib. However, their survival benefits are limited, and their clinically relevant toxicities have highlighted the need for more effective and better tolerated treatment strategies (3, 4).

Immune checkpoint inhibitors (ICIs) are designed to restore antitumour T-cell activity within the immunosuppressive liver tumour microenvironment. Because phase III trials of ICI monotherapy did not consistently show a survival benefit, clinical development has moved toward combination strategies (5). These strategies usually combine ICIs with anti-angiogenic agents to support vascular normalisation or use dual checkpoint blockade to improve T-cell priming and effector function (6, 7). However, treatment outcomes have been inconsistent across trials. Atezolizumab plus bevacizumab established a new standard of care in IMbrave150, with a median overall survival (OS) of 19.2 months. Positive phase III results were also reported for the STRIDE regimen in HIMALAYA and for camrelizumab plus rivoceranib in CARES-310 (8–10). In contrast, other combinations failed to meet prespecified superiority thresholds, as shown in LEAP-002, or improved progression-free survival (PFS) without a clear OS benefit, as shown in COSMIC-312 (11, 12). These mixed results show the need for a systematic synthesis that can separate evidence for broad therapeutic strategies from evidence for individual regimens. Such an approach may provide a clearer and more clinically useful interpretation of the current evidence.

In this study, we performed an updated Bayesian network meta-analysis (NMA) of phase III randomised trials evaluating first-line ICI-based systemic therapy for advanced HCC. In addition to conventional regimen-level comparisons, we used a dual-level analytical framework. Treatments were first grouped according to their main mechanistic strategy and were then analysed as individual regimens. This approach allows strategy-level evidence to be considered together with regimen-level findings. It also helps identify where class-level conclusions are supported and where interpretation should remain specific to individual regimens. By jointly evaluating efficacy, safety, treatment discontinuation, and exploratory subgroup effects, this framework provides a structured assessment of the current evidence base for first-line immunotherapy. It is not intended to replace guideline-based treatment selection. Instead, it serves as an evidence-mapping tool that distinguishes class-level signals from regimen-specific findings and clarifies where evidence is consistent and where greater caution is needed.

## 2 Methods

This NMA was conducted in accordance with the Preferred Reporting Items for Systematic Reviews and Meta-Analyses and its extension for network meta-analyses (PRISMA-NMA) (13, 14). The protocol was prospectively registered in PROSPERO (CRD420251273309). Ethics approval and informed consent were not required because only previously published aggregate data were used.

### 2.1 Search strategy and study selection

We searched PubMed (MEDLINE), Embase, the Cochrane Library, and the Web of Science Core Collection from inception to 28 December 2025. Targeted searches of major conference proceedings, including those of the American Society of Clinical Oncology (ASCO) and the European Society for Medical Oncology (ESMO), were also performed to identify recently reported phase III trials and long-term follow-up updates. The complete search strategies are provided in **Supplementary Table 1**.

Randomised phase III trials were eligible if they met both of the following criteria: (1) they evaluated at least one ICI-based regimen, including ICI monotherapy or an ICI-containing combination, against an active systemic control; and (2) they were conducted in the first-line setting for advanced or unresectable HCC. Trials were excluded if they were phase I/II, non-randomised, or single-arm studies; if they were restricted to intermediate-stage disease eligible for locoregional therapy; or if they evaluated systemic therapy in mandatory combination with locoregional treatment. When trials were reported in multiple publications or updated analyses, all available reports were reviewed together, and the most complete and most recent data were extracted to avoid double counting.

### 2.2 Data extraction and risk of bias assessment

Two investigators independently screened the records and extracted data. Disagreements were resolved by consensus or by adjudication from a third investigator. The primary outcomes were OS and PFS. Secondary outcomes included objective response rate (ORR) and grade ≥3 treatment-related adverse events (TRAEs). Risk of bias was independently assessed by two investigators using the original Cochrane Collaboration Risk of Bias tool. The assessed domains included random sequence generation, allocation concealment, blinding of participants and personnel, blinding of outcome assessment, incomplete outcome data, selective reporting, and other bias. Each domain was rated as having a low, unclear, or high risk of bias (15).

### 2.3 Assessment of transitivity and follow-up maturity

To assess the plausibility of transitivity, we compared the distribution of prespecified effect modifiers across the main strategy comparison classes. These effect modifiers included viral aetiology, represented by the proportions of patients with hepatitis B virus (HBV) and hepatitis C virus (HCV) infection; baseline tumour burden, represented by the proportions of patients with macrovascular invasion (MVI) and extrahepatic spread (EHS); hepatic reserve, represented by the proportion of patients with Child-Pugh class A liver function; performance status, represented by the proportion of patients with an Eastern Cooperative Oncology Group (ECOG) performance status of 0; baseline alpha-fetoprotein (AFP), represented by the proportion of patients with AFP ≥400 ng/mL; and OS follow-up maturity, represented by the median follow-up duration where reported.

Distributions were summarised at the trial level and presented by strategy comparison class as medians and interquartile ranges, or as medians and ranges when fewer than three trials were available. Quantitative between-class comparisons were performed using Kruskal-Wallis tests based on trial-level percentages or trial-level follow-up duration. Because several strategy classes included only one or two trials, these tests had limited statistical power. Therefore, they were used to support descriptive inspection rather than to provide definitive evidence of balance or imbalance between classes. Trials with missing values for a given variable were excluded from the corresponding analysis.

### 2.4 Treatment classification and network structure

Because first-line ICI-based therapy for advanced HCC includes multiple regimens with overlapping but non-identical mechanisms, we prespecified a strategy-regimen analytical framework to summarise the evidence at two complementary levels. The strategy-level analysis evaluated broad therapeutic approaches commonly used in clinical decision-making, whereas the regimen-level analysis preserved treatment-specific information and assessed whether class-level patterns were supported by individual regimen estimates. Accordingly, the two analyses were fitted as separate models with different inferential targets: the strategy-level model estimated class-level effects after grouping regimens by dominant therapeutic mechanism, whereas the regimen-level model estimated treatment-specific effects.

Regimens were classified *a priori* into mechanism-based strategy classes: PD-(L)1 monotherapy, PD-(L)1 plus cytotoxic T-lymphocyte-associated protein 4 (CTLA-4) blockade, PD-(L)1 plus tyrosine kinase inhibitor (TKI), PD-(L)1 plus VEGF monoclonal or biosimilar antibody [VEGF(Ab)], and T-cell immunoreceptor with Ig and ITIM domains (TIGIT) plus PD-(L)1 plus VEGF(Ab). TKI monotherapy was used as the common comparator at the strategy level. Classification was based on the dominant treatment modality and clinical regimen structure rather than target overlap alone. Therefore, orally administered small-molecule kinase inhibitors, including VEGFR-selective TKIs, were classified as TKI-based regimens, whereas VEGF-targeting monoclonal or biosimilar antibodies were classified as VEGF(Ab)-based regimens.

At the regimen level, each individual regimen was retained as a separate treatment node, with sorafenib used as the common reference. REFLECT was included to provide the lenvatinib–sorafenib connection for trials using lenvatinib as the control. CheckMate 9DW used an investigator’s-choice TKI control arm consisting of lenvatinib or sorafenib. At the strategy level, this arm was classified as TKI monotherapy and therefore required no decomposition. At the regimen level, because sorafenib was the reference node, the mixed-control arm was incorporated using a prespecified mixture-control approach. In the base-case analysis, the mixture weight was set at (w = 0.85), corresponding to the reported proportion of control-arm patients receiving lenvatinib. Deterministic sensitivity analyses across alternative fixed values of (w) and a probabilistic sensitivity analysis using an allocation-derived Beta prior for (w) were performed to assess the influence of this assumption.

### 2.5 Statistical analysis

Bayesian NMA was performed in R using the gemtc package with JAGS. Consistency models were fitted using Markov chain Monte Carlo sampling with four chains, 20,000 burn-in iterations, and 50,000 sampling iterations per chain, with a thinning interval of 10. Convergence was assessed using trace and density plots and the Gelman–Rubin diagnostic, with a potential scale reduction factor <1.05 considered acceptable (16). Treatment effects were modelled on the log scale and reported as hazard ratios (HRs) for OS and PFS and odds ratios (ORs) for ORR and grade ≥3 TRAEs, with 95% credible intervals (CrIs).

Analyses were performed at both the strategy and regimen levels. At the strategy level, random-effects consistency models were used as the primary analyses, with fixed-effect models fitted for comparison. Between-study heterogeneity was summarised using the posterior median and 95% CrI of the between-study standard deviation τ, and model fit was assessed using the deviance information criterion (DIC). As a complementary frequentist assessment, heterogeneity was evaluated using Cochran’s Q test and quantified with the I² statistic. At the regimen level, fixed-effect consistency models were used as the base-case analyses because of the sparse network structure and the limited number of trials informing several direct comparisons. Random-effects models were fitted as sensitivity analyses. Network inconsistency was assessed where feasible. Because the strategy-level network had limited closed-loop evidence, formal node-splitting was not feasible; therefore, inconsistency assessment was performed by comparing DIC values between consistency and unrelated mean-effects models. At the regimen level, formal inconsistency assessment was further limited by network sparsity. For each endpoint, treatment ranking was summarised using the surface under the cumulative ranking curve (SUCRA) and the posterior probability of being ranked first, denoted Pr(rank = 1). SUCRA summarises the overall cumulative ranking profile, whereas Pr(rank = 1) focuses on the probability of occupying the first rank and is more sensitive to sparse evidence and imprecise estimates.

To explore potential OS effect modification by aetiology and baseline tumour burden, within-trial ratios of hazard ratios (RHRs) were calculated from published subgroup-specific HRs. Subgroup contrasts included HBV-related versus non-viral HCC, HCV-related versus non-viral HCC, and MVI and/or EHS versus neither MVI nor EHS. RHR was defined as the HR in the first subgroup divided by the HR in the reference subgroup, with RHR <1 indicating a greater relative treatment effect in the first subgroup. When at least two trials reported the same subgroup contrast for a comparable strategy class, RHRs were pooled using inverse-variance random-effects meta-analysis and reported with 95% confidence intervals (CIs).

## 3 Results

### 3.1 Study selection and characteristics

The systematic literature search identified 3,422 records. After deduplication and screening, 15 phase III randomised controlled trials (RCTs) involving 9,792 patients with advanced HCC were included in the quantitative synthesis (**Supplementary Figure 1**). Thirteen trials were published as full-text articles (3, 5, 8–12, 17–22), whereas two were available only as conference abstracts (23, 24). Overall risk of bias was generally low across the included trials (**Supplementary Figure 2**). Baseline characteristics are summarised in **Supplementary Table 2**.

Across the main strategy comparison classes, the distributions of prespecified trial-level effect modifiers were generally comparable (**Supplementary Table 3**). The proportions of patients with Child-Pugh class A liver function were consistently high across classes, while ECOG performance status 0, MVI, EHS, and AFP ≥400 ng/mL showed overlapping distributions. Greater variation was observed for viral aetiology: trials evaluating PD-(L)1 plus VEGF(Ab) were generally enriched for HBV-related disease, whereas HCV proportions were lower in this class. OS follow-up maturity also varied across strategy classes, largely reflecting differences in reporting time points and analysis maturity. Exploratory Kruskal-Wallis tests did not detect statistically significant between-class differences in HBV infection, HCV infection, MVI, EHS, Child-Pugh class A liver function, ECOG performance status 0, AFP ≥400 ng/mL, or median OS follow-up duration. Overall, these findings did not suggest major imbalances in measured trial-level effect modifiers. However, viral aetiology and follow-up maturity remained important contextual factors for interpretation.

**Figure 1:**
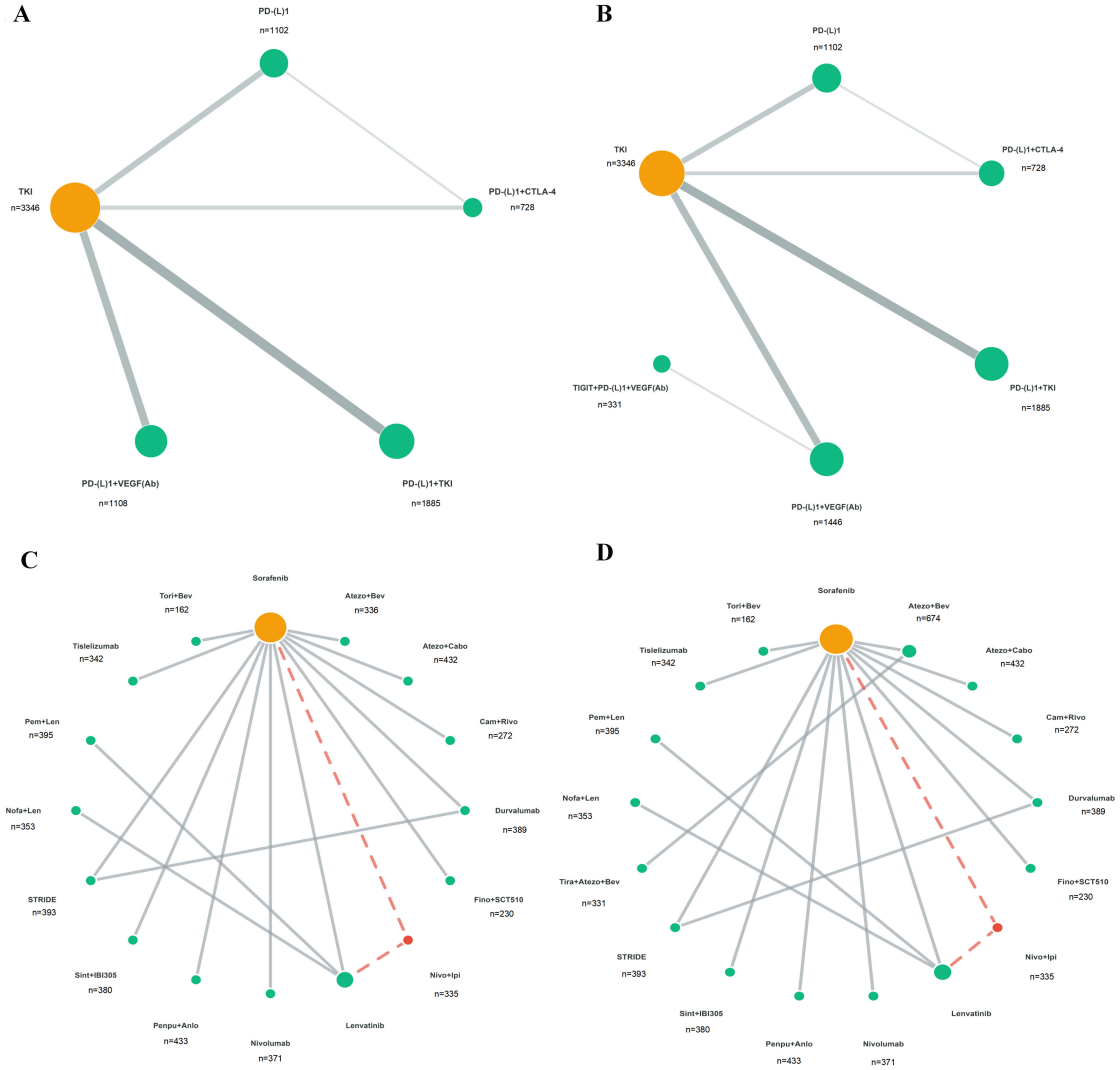
Evidence network geometry for first-line ICI-based therapies. **(A)** Strategy-level network for overall survival (OS), anchored on tyrosine kinase inhibitor (TKI) control. **(B)** Strategy-level network for progression-free survival (PFS), anchored on TKI control. **(C)** Regimen-level network for OS, anchored on sorafenib. **(D)** Regimen-level network for PFS, anchored on sorafenib. Nodes represent strategy classes **(A, B)** or individual regimens **(C, D)**; node size is proportional to the number of trials contributing to each node. Edges indicate direct randomised comparisons; edge thickness is proportional to the number of events informing each direct comparison. ICI, immune checkpoint inhibitor; OS, overall survival; PFS, progression-free survival; TKI, tyrosine kinase inhibitor; PD-(L)1, programmed death-1/programmed death-ligand 1; CTLA-4, cytotoxic T-lymphocyte-associated protein 4; VEGF(Ab), VEGF monoclonal antibody; TIGIT, T-cell immunoreceptor with Ig and ITIM domains; Atezo+Bev, atezolizumab plus bevacizumab; Atezo+Cabo, atezolizumab plus cabozantinib; Cam+Rivo, camrelizumab plus rivoceranib; Fino+SCT510, finotonlimab plus SCT510; Pem+Len, pembrolizumab plus lenvatinib; Sinti+IBI305, sintilimab plus IBI305; STRIDE, Single Tremelimumab Regular Interval Durvalumab; Tira+Atezo+Bev, tiragolumab plus atezolizumab and bevacizumab; Tori+Bev, toripalimab plus bevacizumab; Nofa+Len, nofazinlimab plus lenvatinib; Nivo+Ipi, nivolumab plus ipilimumab.

The strategy-level network compared mechanism-based classes against TKI monotherapy and included four PD-(L)1 plus VEGF(Ab) trials, five PD-(L)1 plus TKI trials, two PD-(L)1 plus CTLA-4 trials, and two PD-(L)1 monotherapy trials (**Figures 1A, B**). IMbrave152 evaluated a TIGIT-containing strategy but was excluded from the OS network because OS data were immature.

The regimen-level network was anchored to sorafenib (**Figures 1C, D**). CheckMate 9DW used an investigator’s-choice TKI control arm, in which investigators selected either lenvatinib or sorafenib before treatment allocation. For the regimen-level analysis, this mixed-control arm was incorporated using a prespecified mixture-control approach. The base-case mixture weight was set at (w = 0.85), corresponding to the actual composition of the CheckMate 9DW control arm, in which 275 of 325 patients (85%) received lenvatinib and 50 of 325 patients (15%) received sorafenib. Thus, the mixture weight reflected the within-trial investigator-selected control distribution rather than an external assumption.

### 3.2 Model fit, heterogeneity, and consistency assessment

All Bayesian models showed satisfactory convergence, with potential scale reduction factors close to 1.00 across endpoints. Model fit and heterogeneity are summarised in **Supplementary Table 4**. At the strategy level, random-effects models were used as the primary analyses because they generally provided better model fit for PFS, ORR, and grade ≥3 TRAEs, although OS showed similar fit between fixed-effect and random-effects models. The random-effects DIC values were 21.06 for OS, 27.23 for PFS, 55.15 for ORR, and 56.45 for grade ≥3 TRAEs, with corresponding posterior median τ values of 0.05, 0.12, 0.41, and 0.34, respectively. Complementary frequentist analyses showed total network I² values of 15%, 54%, 66%, and 70% for OS, PFS, ORR, and grade ≥3 TRAEs, respectively, with within-design heterogeneity P values of 0.170, 0.005, <0.001, and <0.001.

At the regimen level, fixed-effect models were used as the base-case analyses for OS and PFS because the network was sparse and several comparisons were informed by single trials. Random-effects models did not materially improve model fit, with fixed-effect versus random-effects DIC values of −10.18 versus −10.42 for OS and −11.61 versus −11.44 for PFS; therefore, regimen-level random-effects models were interpreted as sensitivity analyses.

Strategy-level consistency assessment showed no evidence of meaningful global inconsistency, as consistency and unrelated mean-effects models had similar DIC values across endpoints, with ΔDIC values close to zero (**Supplementary Table 5**).

### 3.3 Strategy-level NMA and treatment ranking

**Figure 2:**
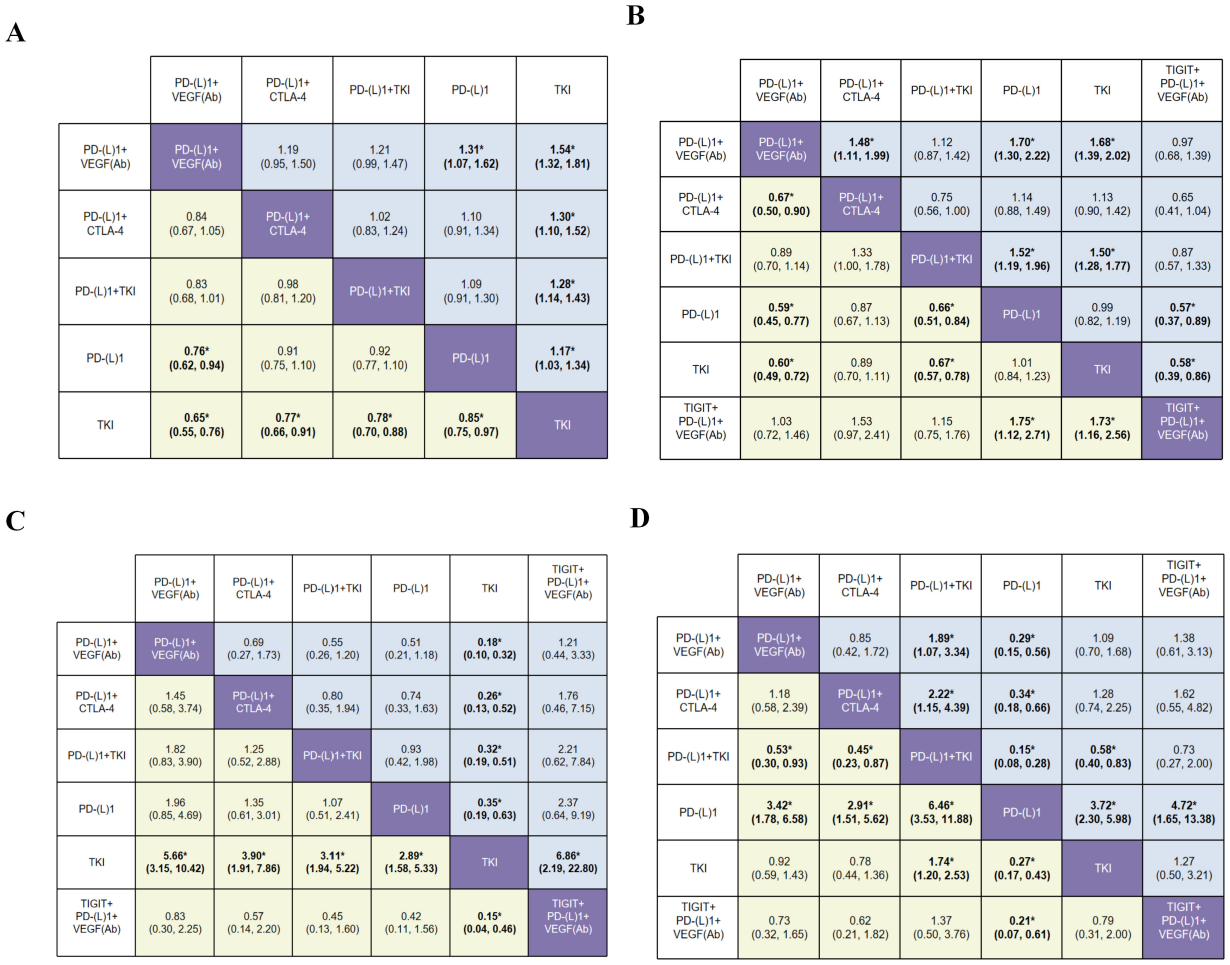
Strategy-level league tables for efficacy and safety. **(A)** OS. **(B)** PFS. **(C)** ORR. **(D)** Grade ≥3 TRAEs. Each cell shows the posterior median effect with 95% CrI comparing the column strategy with the row strategy (column/row). Effect measures were HRs for OS and PFS and ORs for ORR and grade ≥3 TRAEs. For OS, PFS, and grade ≥3 TRAEs, values <1 favour the column strategy; for ORR, values >1 favour the column strategy. Asterisks indicate comparisons for which the 95% CrI excluded the null value of 1.00. CrI, credible interval; HR, hazard ratio; OR, odds ratio; ORR, objective response rate; TRAE, treatment-related adverse event.

At the strategy level, PD-(L)1 plus VEGF(Ab) showed the largest OS benefit versus TKI monotherapy (HR 0.65, 95% CrI 0.55–0.76) and had the highest OS SUCRA value (0.98) (**Figure 2A**). PD-(L)1 plus CTLA-4 (HR 0.77, 95% CrI 0.66–0.91) and PD-(L)1 plus TKI (HR 0.78, 95% CrI 0.70–0.88) also showed evidence of OS benefit, whereas PD-(L)1 monotherapy had a smaller effect estimate (HR 0.85, 95% CrI 0.75–0.97). For PFS, PD-(L)1 plus VEGF(Ab) also had the highest SUCRA value (HR 0.60, 95% CrI 0.49–0.72; SUCRA 0.85), followed by PD-(L)1 plus TKI (HR 0.67, 95% CrI 0.57–0.78) (**Figure 2B**). ORR was highest with PD-(L)1 plus VEGF(Ab) (OR 5.66, 95% CrI 3.15–10.42) (**Figure 2C**). The TIGIT-containing strategy ranked highly for PFS and ORR, but estimates were imprecise because evidence was derived from a single trial with wide CrIs (**Supplementary Figure 3**).

For safety, PD-(L)1 monotherapy was the most favourable strategy for grade ≥3 TRAEs (SUCRA 1.00) and had lower odds of grade ≥3 TRAEs than TKI monotherapy (OR 0.27, 95% CrI 0.17–0.43) (**Figure 2D**). Among combination strategies, PD-(L)1 plus TKI had the highest burden of grade ≥3 TRAEs (OR 1.74, 95% CrI 1.20–2.53) and the least favourable tolerability ranking (SUCRA 0.06). No clear differences in grade ≥3 TRAEs were observed for PD-(L)1 plus VEGF(Ab) or PD-(L)1 plus CTLA-4 compared with TKI monotherapy. To ensure consistency in ranking interpretation across analytical levels, we additionally calculated the posterior probability of being ranked first, Pr(rank = 1), at the strategy level. These probabilities generally supported the SUCRA-based hierarchy and are summarised in **Supplementary Table 6**.

### 3.4 Regimen-level performance

**Figure 3:**
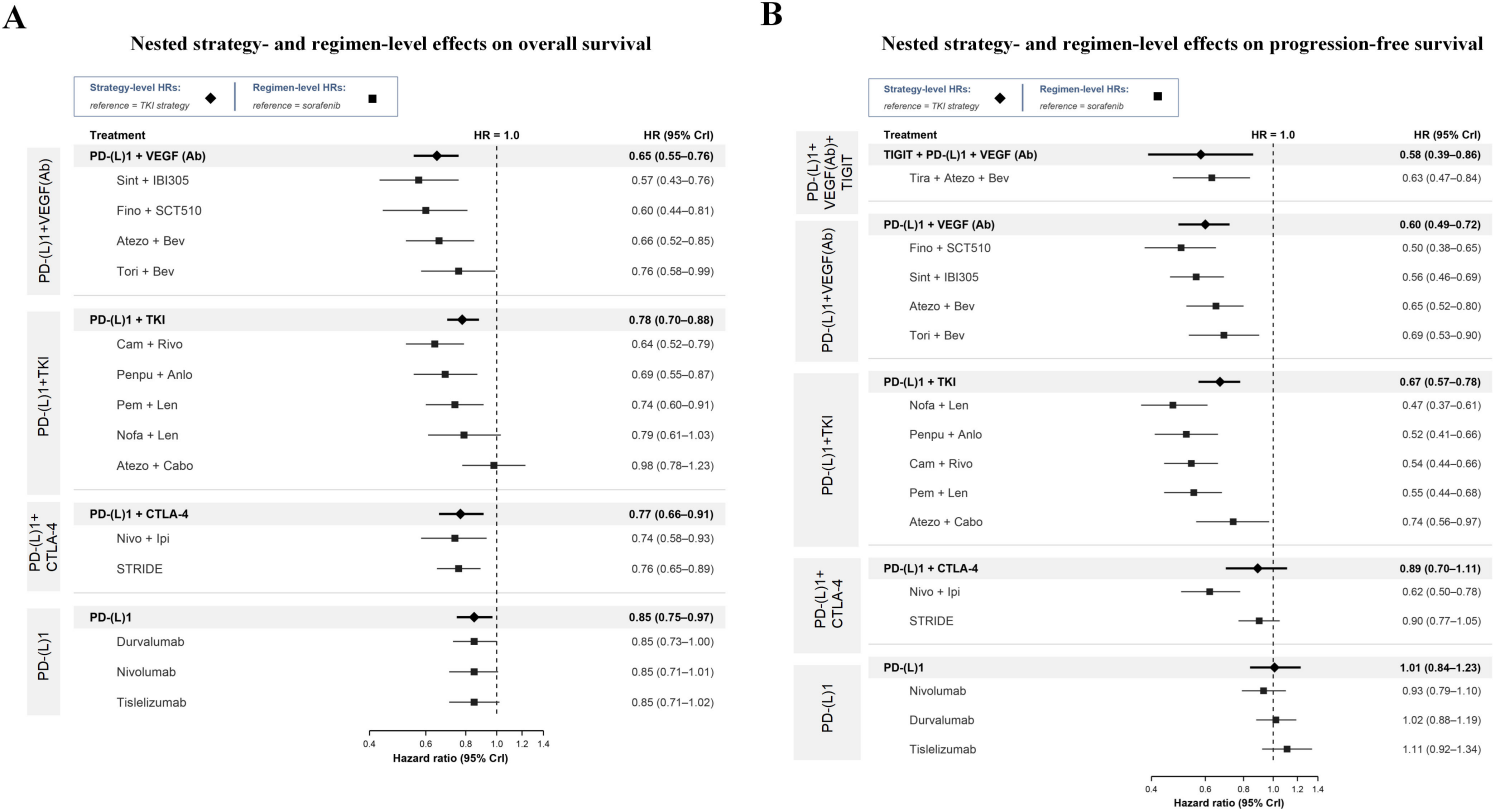
Nested forest plots of strategy- and regimen-level effects. **(A)** OS. **(B)** PFS. Diamonds indicate strategy-level HRs versus TKI, and squares indicate regimen-level HRs versus sorafenib. The vertical line indicates the no-effect reference at HR = 1.

Regimen-level estimates generally followed the strategy-level hierarchy, but important differences were observed within some strategy classes. The nested forest plot shows individual regimen effects within each mechanistic strategy class. For OS, several combinations showed evidence of benefit versus sorafenib, including atezolizumab plus bevacizumab (HR 0.66, 95% CrI 0.52–0.85), sintilimab plus IBI305 (HR 0.57, 95% CrI 0.43–0.76), camrelizumab plus rivoceranib (HR 0.64, 95% CrI 0.52–0.79), and tremelimumab plus durvalumab (STRIDE) (HR 0.76, 95% CrI 0.65–0.89) (**Figure 3A**). For PFS, several combination regimens also showed evidence of benefit, including camrelizumab plus rivoceranib (HR 0.54, 95% CrI 0.44–0.66) and atezolizumab plus bevacizumab (HR 0.65, 95% CrI 0.52–0.80) (**Figure 3B**). The SUCRA-based ranking profiles are provided in **Supplementary Table 7**.

As an additional exploratory analysis, we calculated the posterior probability that each regimen ranked first within its corresponding strategy class. The rank-probability profiles showed heterogeneity within several strategy classes, with different regimens having the highest Pr(rank = 1) for OS and PFS (**Supplementary Figure 4**). These within-class ranking metrics were interpreted descriptively because several regimen-level estimates were informed by sparse or single-trial evidence.

The range of regimen-level OS estimates varied across strategy classes. PD-(L)1 plus VEGF(Ab) showed a relatively narrow OS HR range (0.57–0.76), suggesting consistent effects across available VEGF antibody-based regimens. PD-(L)1 plus TKI showed a broader range of OS HRs (0.64–0.98), indicating that regimen-level interpretation remains important for this class. For PD-(L)1 plus CTLA-4 and PD-(L)1 monotherapy, the limited number of trials precluded a robust assessment of within-class variation.

### 3.5 Benefit-risk profile and selected TRAEs

**Figure 4:**
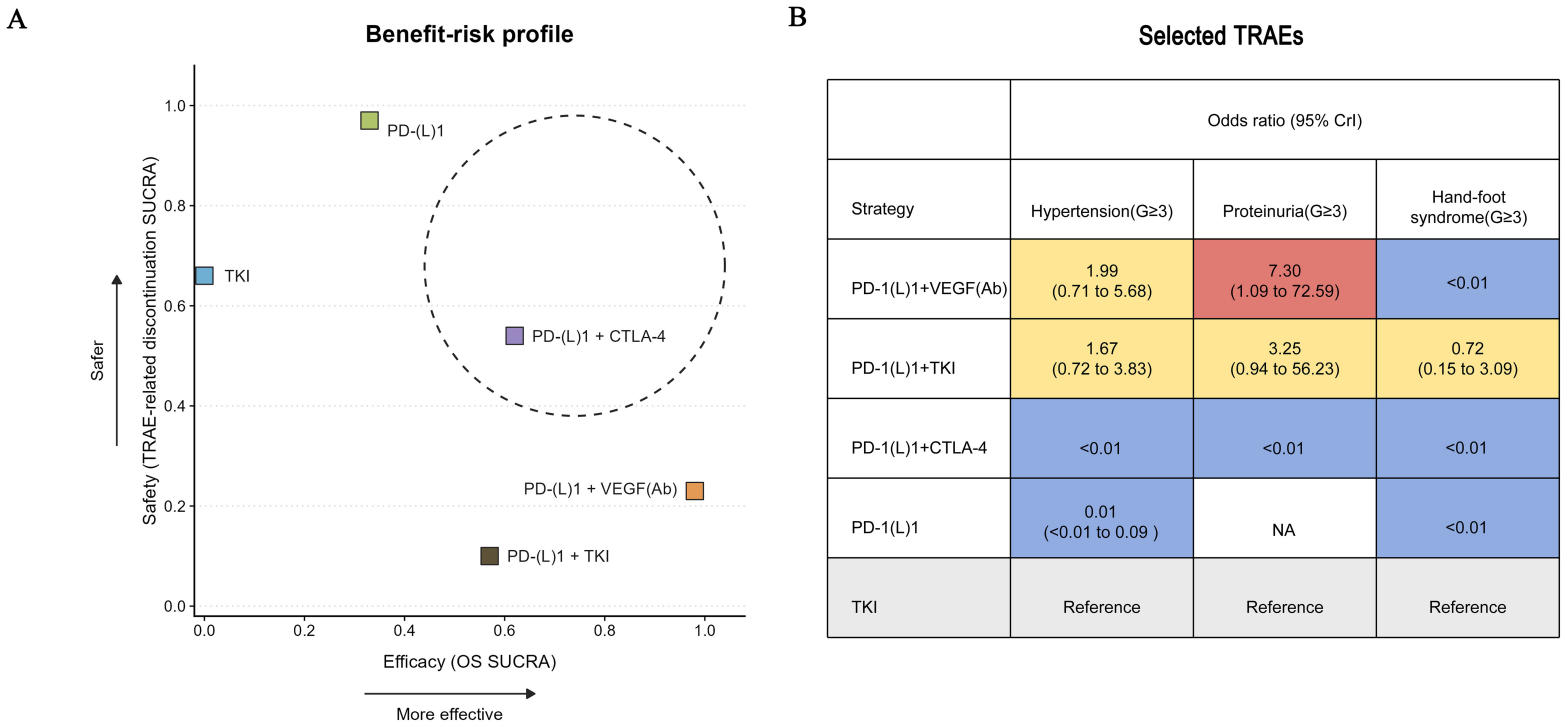
Benefit-risk profile of strategy classes. **(A)** Benefit-risk profile based on SUCRA values for OS and TRAE-related discontinuation. Higher OS SUCRA indicates a more favourable efficacy ranking, and higher discontinuation SUCRA indicates a more favourable safety ranking with lower relative discontinuation burden. **(B)** Selected grade ≥3 TRAEs, expressed as OR versus TKI.

The benefit-risk profile of each strategy was further assessed by plotting OS SUCRA against SUCRA for TRAE-related treatment discontinuation. Higher values indicated more favourable rankings for efficacy or safety (**Figure 4A**). PD-(L)1 plus VEGF(Ab) showed the most favourable OS ranking, but its ranking for TRAE-related discontinuation was less favourable (SUCRA 0.23). PD-(L)1 monotherapy showed the most favourable discontinuation profile (SUCRA 0.97), although its OS ranking was lower. PD-(L)1 plus CTLA-4 occupied an intermediate position for both OS and TRAE-related discontinuation (SUCRA 0.54), suggesting a relatively balanced profile. In contrast, PD-(L)1 plus TKI showed intermediate OS ranking but the least favourable ranking for TRAE-related discontinuation (SUCRA 0.10).

To characterise class-defining toxicities, we compared grade ≥3 hypertension, proteinuria, and hand-foot syndrome across strategy classes (**Figure 4B**). PD-(L)1 plus VEGF(Ab) was associated with a higher odds of grade ≥3 proteinuria compared with TKI monotherapy (OR 7.30, 95% CrI 1.09–72.59), although the interval was wide. No clear differences were observed for grade ≥3 hypertension, proteinuria, or hand-foot syndrome with PD-(L)1 plus TKI compared with TKI monotherapy. PD-(L)1 plus CTLA-4 and PD-(L)1 monotherapy showed low odds for these selected anti-angiogenic or TKI-associated toxicities. Grade ≥3 immune-related adverse events (irAEs) were inconsistently reported across trials and were therefore summarised descriptively in ICI-containing arms (**Supplementary Table 8**). The weighted incidence was highest with PD-(L)1 plus CTLA-4 (19.7%; 95% CI 17.0–22.8%; 2 trials), followed by PD-(L)1 plus TKI (9.8%; 95% CI 8.2–11.7%; 3 trials), PD-(L)1 plus VEGF(Ab) (9.7%; 95% CI 7.1–13.0%; 2 trials), and PD-(L)1 monotherapy (7.3%; 95% CI 5.6–9.4%; 2 trials). Detailed regimen-level estimates for grade ≥3 TRAEs and additional adverse events are shown in **Supplementary Figure 5**.

### 3.6 Subgroup analyses

**Figure 5:**
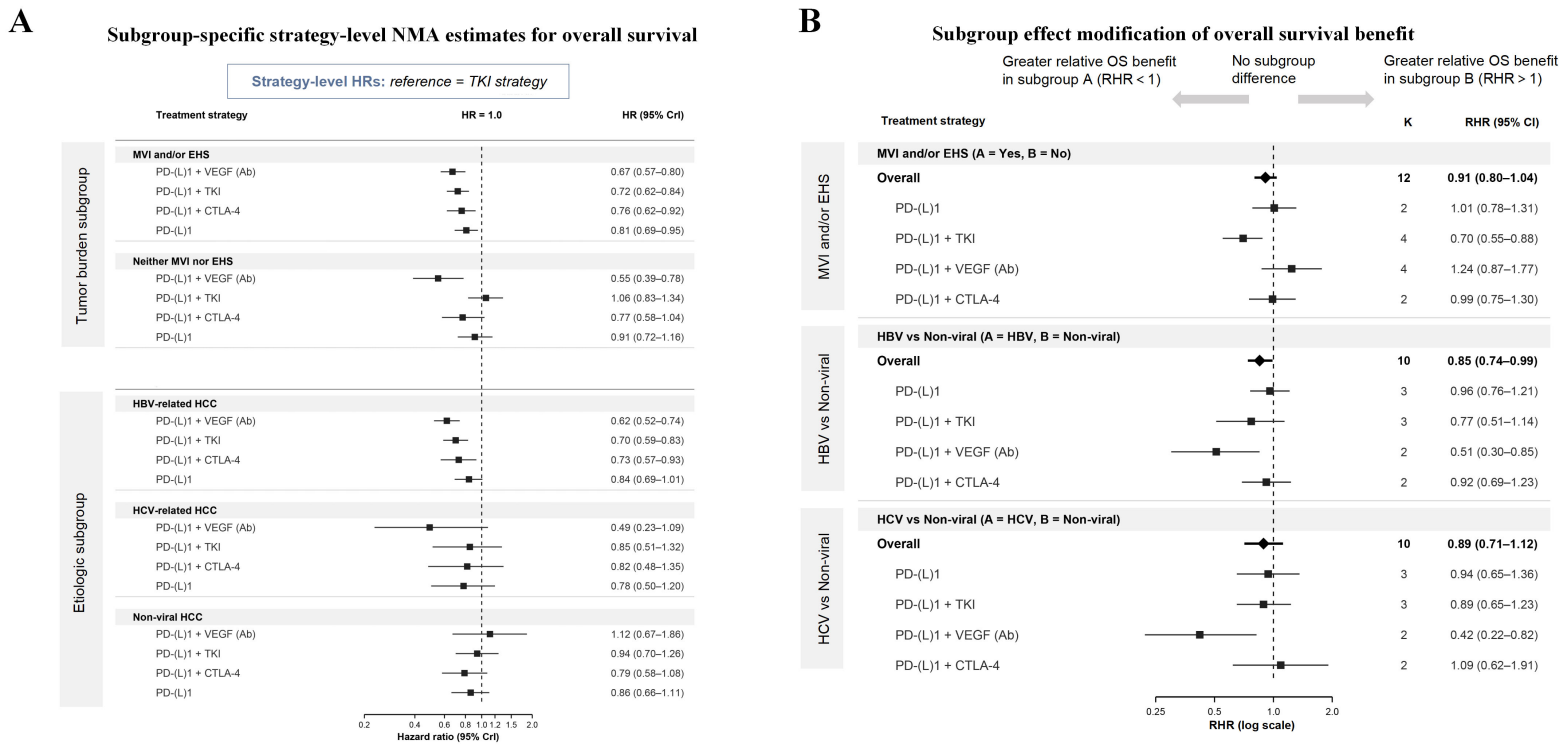
Subgroup-specific strategy-level network meta-analysis and effect-modification analysis for overall survival. **(A)** Subgroup-specific strategy-level NMA estimates comparing each ICI-based strategy with the TKI strategy within each patient subgroup. **(B)** Within-trial ratios of hazard ratios (RHRs) assessing potential effect modification between paired subgroups. RHR = HR (subgroup A)/HR (subgroup B). RHR <1 indicates that subgroup A benefits more; RHR >1 indicates that subgroup B benefits more.

Subgroup analyses were conducted using subgroup-specific NMAs and within-trial RHR analyses. In the subgroup-specific NMAs, the overall strategy-level hierarchy was broadly preserved among patients with MVI and/or EHS. In this subgroup, all ICI-based strategies were associated with improved OS compared with the TKI strategy, including PD-(L)1 plus VEGF(Ab) (HR 0.67, 95% CrI 0.57–0.80), PD-(L)1 plus TKI (HR 0.72, 95% CrI 0.62–0.84), PD-(L)1 plus CTLA-4 (HR 0.76, 95% CrI 0.62–0.92), and PD-(L)1 monotherapy (HR 0.81, 95% CrI 0.69–0.95) (**Figure 5A**). Among patients with neither MVI nor EHS, PD-(L)1 plus VEGF(Ab) remained associated with improved OS, whereas estimates for the other ICI-based strategies were less definitive.

In aetiology-specific NMAs, several ICI-based strategies were associated with improved OS compared with the TKI strategy in HBV-related HCC, including PD-(L)1 plus VEGF(Ab) (HR 0.62, 95% CrI 0.52–0.74), PD-(L)1 plus TKI (HR 0.70, 95% CrI 0.59–0.83), and PD-(L)1 plus CTLA-4 (HR 0.73, 95% CrI 0.57–0.93). By contrast, estimates in HCV-related and non-viral HCC were generally imprecise, with wide CrIs limiting firm conclusions regarding aetiology-specific treatment effects.

Within-trial RHR analyses did not show clear overall effect modification by MVI/EHS status (RHR 0.91, 95% CI 0.80–1.04; k = 12). In strategy-specific analyses, PD-(L)1 plus TKI showed a potential signal of greater relative OS benefit among patients with MVI and/or EHS than among those without these features (RHR 0.70, 95% CI 0.55–0.88; k = 4), which was directionally consistent with the subgroup-specific NMA (**Figure 5B**).

For etiology, the overall RHR suggested a modestly greater relative OS benefit in HBV-related than in non-viral HCC (RHR 0.85, 95% CI 0.74–0.99; k = 10), whereas no clear difference was observed between HCV-related and non-viral HCC (RHR 0.89, 95% CI 0.71–1.12; k = 10). Strategy-specific RHR estimates for PD-(L)1 plus VEGF(Ab) appeared more favorable in HBV-related and HCV-related HCC than in non-viral HCC, but these estimates were each based on only two trials and should therefore be interpreted as hypothesis-generating rather than as evidence for etiology-guided treatment selection.

### 3.7 Sensitivity analysis

Leave-one-out analyses showed that the main NMA estimates were robust to the exclusion of individual studies, with only small changes in effect estimates and stable model fit for both OS and PFS (**Supplementary Figures 6, S7**). The mixed-control assumption for the CheckMate 9DW investigator’s-choice arm was also supported by probabilistic and deterministic sensitivity analyses of the mixture weight, which yielded results consistent with the primary fixed weight of w = 0.85 analysis (**Supplementary Table 9**).

Additional sensitivity analyses examining key modelling and classification assumptions did not materially alter the main findings. These included reassigning camrelizumab plus rivoceranib to the PD-(L)1 plus VEGF(Ab) strategy, excluding IMbrave152, excluding conference abstracts, applying strategy-level fixed-effect models, and applying regimen-level random-effects models (**Supplementary Tables 10–S14**). Overall, the primary NMA findings were robust across these sensitivity analyses.

## 4 Discussion

Previous NMAs of first-line systemic therapy for advanced HCC have mainly provided regimen-level rankings. However, as first-line treatment options continue to expand, clinicians must decide not only which individual regimen appears to rank highest, but also how these regimens should be interpreted within broader therapeutic strategies. In this study, we used a dual-level Bayesian NMA framework to provide a complementary strategy-regimen perspective for first-line ICI-based therapy. This approach reflects a common clinical reasoning process, in which physicians first consider guideline-supported therapeutic strategies and then individualise treatment according to drug availability, hepatic reserve, comorbidities, bleeding risk, immune-related toxicity risk, and expected tolerability. By separating mechanism-based strategies from the individual regimens nested within them, this framework supports interpretation at both the strategy and regimen levels.

**Figure 6:**
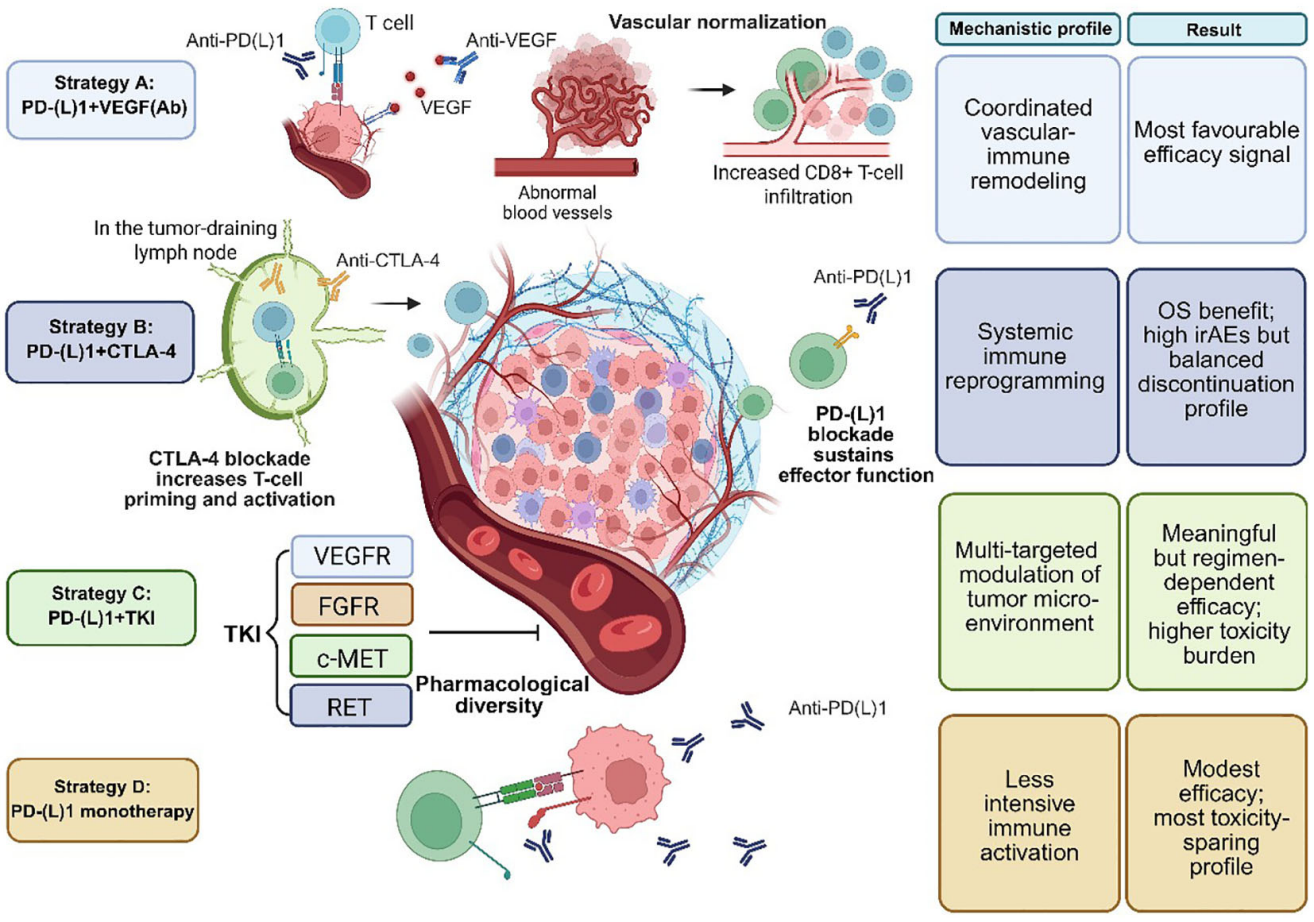
Mechanistic rationale and benefit-risk patterns of first-line ICI-based strategies in advanced hepatocellular carcinoma. PD-(L)1 plus VEGF(Ab) may coordinate vascular normalisation and immune remodelling, supporting its efficacy-oriented profile. PD-(L)1 plus CTLA-4 enhances T-cell priming and activation, producing a distinct immune-based benefit-risk pattern. PD-(L)1 plus TKI combines PD-(L)1 blockade with multi-targeted kinase inhibition, but pharmacological diversity, cumulative toxicity, dose modification, and treatment exposure may contribute to regimen-level heterogeneity. PD-(L)1 monotherapy provides a less intensive immune-based strategy with the most favourable tolerability profile. The image is intended as a conceptual summary and should be interpreted alongside the quantitative NMA results.

The main finding was that PD-(L)1 plus VEGF(Ab) was the most efficacy-oriented strategy and was supported by relatively consistent regimen-level evidence. PD-(L)1 plus TKI showed meaningful antitumour activity but had the least favourable safety and discontinuation profile, indicating a clear efficacy-toxicity trade-off. PD-(L)1 monotherapy was the most tolerable strategy, whereas PD-(L)1 plus CTLA-4 showed an intermediate efficacy-safety profile with a relatively balanced discontinuation burden. Exploratory RHR analyses suggested that tumour burden and viral aetiology may influence the relative OS benefit of ICI-based therapy. Overall, the added value of this dual-level framework lies not only in updating comparative treatment rankings, but also in providing a structured approach to interpret existing evidence according to therapeutic strategy, regimen-level consistency, patient baseline characteristics, and expected toxicity. These strategy-specific benefit–risk patterns and their putative mechanistic underpinnings are summarised schematically in **Figure 6**.

Within this framework, PD-(L)1 plus VEGF(Ab) provided the clearest class-level efficacy signal. This pattern was not attributable to a single outlying regimen, as VEGF antibody-based combinations showed directionally consistent survival benefits at the regimen level. Although this consistency cannot prove transitivity, it supports the view that the strategy-level signal was not driven only by one individual regimen or by marked regimen-level divergence. Biologically, this pattern is consistent with the role of VEGF blockade in modulating the vascular–immune interface of HCC (25). Beyond angiogenesis inhibition, VEGF/VEGFR blockade may enhance antitumour immunity by promoting vascular normalisation, improving tumour perfusion, increasing cytotoxic T-cell infiltration, and supporting a more pro-inflammatory immune microenvironment when combined with PD-1/PD-L1 inhibition (26, 27). Single-cell evidence further suggests that atezolizumab plus bevacizumab may act through complementary immune- and angiogenesis-related mechanisms in advanced HCC (28).

The subgroup findings for PD-(L)1 plus VEGF(Ab) should be interpreted cautiously. In subgroup-specific NMAs, this strategy remained favourable among patients with MVI and/or EHS and among those with HBV-related HCC. However, these subgroups were well represented in the source trials, particularly in VEGF antibody-based studies. Therefore, the agreement between subgroup-specific and overall findings may partly reflect trial composition rather than definitive evidence that these patients uniquely benefit from PD-(L)1 plus VEGF(Ab). The complementary RHR analysis suggested a stronger relative OS benefit in HBV-related HCC, which is biologically plausible given the coexistence of chronic inflammation, immune exhaustion, and VEGF-mediated vascular barriers in virus-related HCC (29). Nevertheless, viral aetiology should not be used as a standalone treatment-selection marker on the basis of aggregate-data evidence alone (30). Clinically, the efficacy-oriented profile of PD-(L)1 plus VEGF(Ab) should be balanced against VEGF-related risks, including proteinuria, bleeding risk, portal hypertension, renal function, hepatic reserve, and drug availability (31, 32).

PD-(L)1 plus CTLA-4 showed a distinct immune-based benefit-risk profile. It should not be viewed simply as the most efficacy-oriented or the most toxicity-sparing strategy. Rather, it represents an alternative immune-based option for selected patients. A key observation was that the higher incidence of grade ≥3 irAEs did not appear to translate into a proportionally higher discontinuation burden. This comparison remained descriptive because irAEs were inconsistently reported across trials. Risk also varied by regimen. In CheckMate 9DW, 28% of patients receiving nivolumab plus ipilimumab experienced grade 3–4 irAEs. By contrast, the single-dose tremelimumab regimen in HIMALAYA was associated with a lower rate of grade 3–4 immune-mediated events. This difference may reflect CTLA-4 dosing intensity and the nature of the toxicities. Immune-mediated toxicities are often temporally clustered and clinically manageable. In contrast, anti-angiogenic or TKI-related toxicities are often more chronic and exposure-dependent. Mechanistically, CTLA-4 blockade may broaden antitumour immunity by enhancing T-cell priming, reducing regulatory T-cell-mediated suppression, and promoting lymph node-derived T-cell activation and tumour trafficking (33, 34). Thus, PD-(L)1 plus CTLA-4 may be most relevant for patients who are unsuitable for VEGF-targeted therapy but have adequate hepatic reserve and can be closely monitored for immune-related toxicity.

PD-(L)1 plus TKI showed meaningful antitumour activity at the strategy level, but this class required more cautious regimen-level interpretation. Its efficacy signal was accompanied by the highest grade ≥3 TRAE burden among combination strategies and the least favourable profile for TRAE-related discontinuation. Moreover, OS benefit varied across individual PD-(L)1 plus TKI regimens. Therefore, the strategy-level estimate should not be assumed to apply uniformly to all TKI-based combinations. Interpretation of this class should consider the specific TKI partner, toxicity burden, dose modification, treatment sustainability, and the maturity of regimen-level evidence.

This within-class variation is biologically plausible. TKIs are pharmacologically diverse, exposure-dependent agents. Although most have anti-angiogenic activity through VEGF/VEGFR inhibition, they differ substantially in their activity against FGFR, MET, AXL, RET, KIT, PDGFR, and other targets. These differences may translate into distinct vascular, immune, and invasion-related effects within the tumour microenvironment (35). For example, lenvatinib has been shown to remodel the HCC immune microenvironment by reducing immunosuppressive infiltrates and enriching T-cell and interferon-related signatures (36). Mechanistically, its FGFR inhibition may restore IFN-γ-inducible chemokines such as CXCL10 (37). Cabozantinib may enhance anti-PD-1 activity through neutrophil recruitment and activation, whereas anlotinib has been linked to VEGFR2/AKT/HIF-1α/TFRC-related regulation of CD8+ T-cell recruitment (38, 39). These observations suggest that different TKIs may enhance anti-PD-(L)1 therapy through pharmacologically distinct mechanisms. Therefore, TKI-based combinations within this strategy class should not be considered biologically equivalent.

The toxicity findings further support this regimen-level interpretation. In our analysis, PD-(L)1 plus TKI was associated with the highest overall grade ≥3 TRAE burden among combination strategies. However, selected class-defining toxicities, including grade ≥3 hypertension, proteinuria, and hand-foot syndrome, were not clearly higher than with TKI monotherapy. This suggests that the main limitation of this strategy is not a single dominant adverse event. Rather, it may lie in the cumulative burden of overlapping toxicities. These toxicities may lead to dose reduction, interruption, or discontinuation. This issue is clinically important because the anti-angiogenic and immunomodulatory effects of TKIs are likely exposure-sensitive. Insufficient dose intensity may weaken vascular and immune remodelling, whereas excessive toxicity may compromise treatment sustainability. Across phase III PD-(L)1 plus TKI trials, dose reductions in combination arms ranged from approximately 16% to 67%. TRAE-related treatment discontinuation ranged from approximately 6% to 24%. These findings suggest that maintaining adequate dose intensity may partly determine whether early disease control translates into durable OS benefit.

Notably, PD-(L)1 plus TKI showed a clearer OS benefit in patients with MVI and/or EHS, a pattern further supported by the within-trial RHR analysis. This signal is biologically plausible because MVI and EHS reflect a more angiogenic, invasive, and growth factor-driven disease state. Single-cell analyses of venous tumour thrombus in HCC have also identified a distinct immunosuppressive niche enriched for specialised macrophage states and chemotaxis-related programmes (40). In this context, selected TKI partners may be relevant by targeting angiogenesis, hypoxia-associated signalling, invasion-related pathways, and immune exclusion. Nevertheless, this finding remains hypothesis-generating because aggregate-data NMA cannot account for kinase inhibition spectra, dose intensity, treatment exposure, or patient-level tumour biology.

PD-(L)1 monotherapy had a more modest efficacy profile but the most favourable tolerability, supporting its use as a de-escalation option for patients unsuitable for combination therapy.

### 4.1 Limitations

Several limitations should be acknowledged. First, the limited network geometry precluded robust node-splitting inconsistency assessment; therefore, indirect comparisons relied mainly on the transitivity assumption. Although measured trial-level effect modifiers did not differ significantly across strategy classes, these comparisons were underpowered, and clinically relevant imbalances may remain. PD-(L)1 plus VEGF(Ab) trials included higher proportions of patients with HBV-related disease and MVI/EHS and had shorter median OS follow-up than the PD-(L)1 plus CTLA-4 or PD-(L)1 plus TKI classes. These differences could have confounded indirect comparisons and should be considered when interpreting the strategy-level hierarchy. Similarly, the consistency between subgroup-specific and overall findings may partly reflect the composition of the source trials rather than independent confirmation of subgroup-specific treatment effects.

Second, all analyses were based on published aggregate data. We could not adjust for individual-level factors such as geographic region, post-progression therapy, hepatic reserve, AFP level, MVI/EHS status, viral aetiology, PD-L1 expression, tumour mutational burden, or other biomarkers. Mechanistic interpretations regarding VEGF blockade, CTLA-4 inhibition, and TKI-based combinations should therefore be viewed as biologically plausible explanations rather than mechanisms formally tested by this NMA. Subgroup-specific NMAs and RHR analyses were also exploratory because subgroup definitions and reporting were not standardised across trials.

Third, the regimen-level network was sparse, and several comparisons were informed by single trials. Fixed-effect models were used as the base-case regimen-level analyses, but this may have underestimated uncertainty around regimen-specific estimates. Random-effects sensitivity analyses produced broadly consistent treatment hierarchies but wider credible intervals. Accordingly, regimen-level rankings and within-class Pr(rank = 1) should be interpreted as exploratory descriptive metrics rather than definitive evidence of regimen superiority.

Fourth, several modelling and evidence-maturity assumptions should be noted. The CheckMate 9DW mixed-control arm was modelled according to the reported lenvatinib/sorafenib allocation. Stochastic prior-sensitivity analyses for the mixture weight and deterministic scans across fixed weight values yielded stable OS and PFS estimates. IMbrave152 was included in PFS, ORR, and safety analyses because it is the only phase III trial of a first-line TIGIT-containing strategy in advanced HCC. However, its immature OS data were excluded from the OS network, and TIGIT-related findings were interpreted as exploratory. Leave-one-out analyses, including exclusion of IMbrave152 where applicable, did not materially alter the main conclusions. Sensitivity analyses excluding the two conference-abstract trials also supported the robustness of the main findings.

Finally, safety reporting was inconsistent across trials. This was particularly evident for irAEs, dose reductions, treatment interruptions, and TRAE-related discontinuation. These inconsistencies limited formal comparative safety assessment. They also precluded reliable estimation of the excess immune-related toxicity associated with PD-(L)1 plus CTLA-4 relative to other strategies.

## 5 Conclusion

In conclusion, this dual-level Bayesian NMA provides a strategy-regimen framework for interpreting first-line ICI-based therapy in advanced HCC. PD-(L)1 plus VEGF(Ab) showed the most consistent efficacy-oriented profile within the current evidence network, whereas PD-(L)1 plus TKI required closer regimen-level interpretation because of greater within-class variation, higher toxicity burden, dose modification, and treatment sustainability concerns. PD-(L)1 plus CTLA-4 represented a distinct immune-based option with OS benefit and a relatively balanced discontinuation profile, while PD-(L)1 monotherapy remained the most tolerable strategy. Overall, efficacy rankings should be interpreted together with toxicity profile, regimen-level evidence, hepatic reserve, tumour burden, viral aetiology, comorbidities, and patient preference. This framework should be viewed as a supplementary evidence-synthesis tool rather than a prescriptive treatment algorithm. Future studies with mature randomised data, individual patient data, and translational validation are needed to clarify the role of TIGIT-containing strategies, validate the exploratory signals identified here, and refine biologically informed treatment selection.

## Data Availability

The original contributions presented in the study are included in the article/**Supplementary Material**. Further inquiries can be directed to the corresponding author.
