# Supplementary material for: A dual-level Bayesian network meta-analysis of first-line immunotherapy in advanced hepatocellular carcinoma: strategy-level and regimen-level evidence
Source: Front Immunol. 2026 Aug 5;17:1907830. doi: 10.3389/fimmu.2026.1907830 (PMC13485808; doi:10.3389/fimmu.2026.1907830)
Supplement: Supplementary file 1 [file DataSheet1.docx]

 **Supplementary Figure S1. PRISMA flow diagram of study selection.**

**Supplementary Figure S2. Risk-of-bias graph for randomized controlled trials.**

**Supplementary Figure S3. Strategy-level SUCRA heatmap across outcomes.**

Heatmap summarising strategy-level SUCRA values (%) from the random-effects Bayesian network meta-analysis for OS, PFS, ORR and toxicity. Values range from 0% to 100%, with higher SUCRA indicating a more favourable ranking. For efficacy outcomes (OS and PFS), rankings were oriented so that lower HRs correspond to higher SUCRA; for ORR, higher response corresponds to higher SUCRA; and for toxicity outcomes, higher SUCRA indicates greater safety (lower toxicity burden). Grey cells indicate outcomes not available/not estimable for a given strategy. SUCRA, surface under the cumulative ranking curve; OS, overall survival; PFS, progression-free survival; ORR, objective response rate; TKI, tyrosine kinase inhibitor; VEGF(Ab), VEGF monoclonal antibody.

**Supplementary Figure S4. Exploratory within-class posterior probabilities of ranking first in the regimen-level network meta-analysis.**

(A) OS. (B) PFS. Bars show the posterior probability that each regimen ranked first among regimens within the same treatment strategy class. Pr(rank = 1), posterior probability of ranking first.

**Supplementary Figure S5. Regimen-level treatment-related adverse events (TRAEs): detailed profile.**

Regimen-level safety outcomes are summarised using odds ratios (ORs) versus the reference control for grade ≥3 TRAEs, serious TRAEs, and TRAE-related discontinuation, as well as specific TRAEs. All safety endpoints shown in this figure are treatment-related. Asterisks (*) denote extreme OR estimates driven by sparse TRAE data (very low event counts and/or zero cells). For visualisation, ORs <0.01 were truncated to 0.01 and ORs >100 were truncated to 100. OR, odds ratio; SAE, serious adverse event; TRAE, treatment-related adverse event.

**Supplementary Figure S6. Leave-one-out (LOO) sensitivity analyses for overall survival (OS).**

(A) Maximum effect-size change (OS). For each iteration, one trial was omitted and the OS network meta-analysis was re-fitted; the plotted value summarises the largest absolute change in the OS effect estimate(s) relative to the primary analysis (all trials included). (B) Change in heterogeneity (OS). LOO analysis showing the impact of omitting each trial on the between-study heterogeneity parameter τ (tau) for OS. (C) Change in SUCRA ranking (OS). LOO analysis showing changes in SUCRA-based rankings for OS after omitting each trial, reflecting the robustness of the OS ranking hierarchy.

**Supplementary Figure S7. Leave-one-out (LOO) sensitivity analyses for progression-free survival (PFS).**

(A) Maximum effect-size change (PFS). For each iteration, one trial was omitted and the PFS network meta-analysis was re-fitted; the plotted value summarises the largest absolute change in the PFS effect estimate(s) relative to the primary analysis (all trials included). (B) Change in heterogeneity (PFS). LOO analysis showing the impact of omitting each trial on the between-study heterogeneity parameter τ (tau) for PFS. (C) Change in SUCRA ranking (PFS). LOO analysis showing changes in SUCRA-based rankings for PFS after omitting each trial, reflecting the robustness of the PFS ranking hierarchy.
