# Supplementary material for: A dual-level Bayesian network meta-analysis of first-line immunotherapy in advanced hepatocellular carcinoma: strategy-level and regimen-level evidence
Source: Front Immunol. 2026 Aug 5;17:1907830. doi: 10.3389/fimmu.2026.1907830 (PMC13485808; doi:10.3389/fimmu.2026.1907830)
Supplement: Supplementary file 2 [file Table1.docx]

**A Dual-Level Bayesian Network Meta-Analysis of First-Line Immunotherapy in Advanced Hepatocellular Carcinoma: Strategy-Level and Regimen-Level Evidence**

**Supplementary Table S1. Database search strategies**

| PubMed search strategy: | 464 articles |
| --- | --- |
| ( ( "Carcinoma, Hepatocellular"[Mesh:NoExp] OR "Liver Neoplasms"[Mesh:NoExp] OR ((hepatocellular[tiab] OR hepatocytic[tiab]) AND (carcinoma[tiab] OR cancer[tiab] OR tumour*[tiab] OR tumor*[tiab] OR neoplasm*[tiab] OR malignan*[tiab])) OR HCC[tiab] OR hepatoma[tiab] ) AND ( "Immune Checkpoint Inhibitors"[Mesh] OR "checkpoint inhibitor*"[tiab] OR "immune checkpoint inhibitor*"[tiab] OR "PD-1"[tiab] OR "PD1"[tiab] OR "PD-L1"[tiab] OR "PDL1"[tiab] OR "CTLA-4"[tiab] OR "CTLA4"[tiab] OR pembrolizumab[tiab] OR nivolumab[tiab] OR atezolizumab[tiab] OR durvalumab[tiab] OR tremelimumab[tiab] OR ipilimumab[tiab] OR sintilimab[tiab] OR camrelizumab[tiab] OR tislelizumab[tiab] OR toripalimab[tiab] OR sugemalimab[tiab] OR penpulimab[tiab] OR serplulimab[tiab] OR envafolimab[tiab] OR zimberelimab[tiab] OR "CS1001"[tiab] OR "H10v"[tiab] OR "SHR-1210"[tiab] OR avelumab[tiab] OR cemiplimab[tiab] OR dostarlimab[tiab] OR retifanlimab[tiab] ) AND ( (advanced[tiab] OR unresectable[tiab] OR non-resectable[tiab] OR inoperable[tiab] OR metastatic[tiab] OR "locally advanced"[tiab] OR "intermediate stage"[tiab] OR "terminal stage"[tiab]) OR ("BCLC"[tiab] AND ("B"[tiab] OR "C"[tiab] OR "advanced"[tiab])) OR ("Stage"[tiab] AND (III[tiab] OR IV[tiab] OR "3"[tiab] OR "4"[tiab] OR "C"[tiab])) ) AND ( "first line"[tiab] OR "first-line"[tiab] OR frontline[tiab] OR "1st line"[tiab] OR "1L"[tiab] OR (first[tiab] AND (line[tiab] OR therapy[tiab] OR treatment[tiab])) OR (previously[tiab] AND (untreated[tiab] OR naive[tiab])) OR "treatment naive"[tiab] OR "treatment-naive"[tiab] OR "therapy naive"[tiab] OR "systemic therap*"[tiab] OR "systemic treatment*"[tiab] ) AND ( "Randomized Controlled Trial"[pt] OR "Controlled Clinical Trial"[pt] OR "Clinical Trial, Phase III"[pt] OR (randomized[tiab] OR randomised[tiab] OR randomization[tiab] OR randomisation[tiab] OR randomly[tiab]) OR ("phase 3"[tiab] OR "phase III"[tiab] OR "phase III"[ot] OR "pivotal trial*"[tiab]) OR "Multicenter Study"[pt:noexp] ) ) NOT ("animals"[mh] NOT "humans"[mh]) | |
| Embase search strategy: | 1666 articles |
| ('carcinoma in the liver':ti,ab,kw OR 'carcinoma of the liver':ti,ab,kw OR 'carcinoma, hepatic cell':ti,ab,kw OR 'carcinoma, hepatocellular':ti,ab,kw OR 'carcinoma, liver':ti,ab,kw OR 'carcinoma, liver cell':ti,ab,kw OR 'hepatic carcinoma':ti,ab,kw OR 'hepatic cell carcinoma':ti,ab,kw OR 'hepato-carcinoma':ti,ab,kw OR 'hepato-cellular carcinoma':ti,ab,kw OR 'hepatocarcinoma':ti,ab,kw OR 'hepatocellular carcinoma':ti,ab,kw OR 'hepatocellular carcinomata':ti,ab,kw OR 'hepatocyte carcinoma':ti,ab,kw OR 'hepatocytic carcinoma':ti,ab,kw OR 'hepatoma':ti,ab,kw OR 'hepatomata':ti,ab,kw OR 'hepatomatous':ti,ab,kw OR 'liver carcinoma':ti,ab,kw OR 'liver carcinoma rupture':ti,ab,kw OR 'malignant hepatoma':ti,ab,kw OR 'primary liver carcinoma':ti,ab,kw OR 'liver cell carcinoma':ti,ab,kw) AND (randomized:ti,ab,kw OR randomised:ti,ab,kw OR randomly:ti,ab,kw OR placebo:ti,ab,kw OR trial:ti,ab,kw OR groups:ti,ab,kw OR 'randomized controlled trial':ti,ab,kw) AND (unresectable:ti,ab,kw OR 'non-resectable':ti,ab,kw OR inoperable:ti,ab,kw OR metastatic:ti,ab,kw OR 'locally advanced':ti,ab,kw OR 'intermediate stage':ti,ab,kw OR advanced:ti,ab,kw OR 'bclc b':ti,ab,kw OR 'bclc c':ti,ab,kw OR 'bclc advanced':ti,ab,kw) AND (immunotherap*:ti,ab,kw OR 'checkpoint inhibitor*':ti,ab,kw OR 'immune checkpoint*':ti,ab,kw OR 'pd 1':ti,ab,kw OR pd1:ti,ab,kw OR 'pd l1':ti,ab,kw OR pdl1:ti,ab,kw OR 'ctla 4':ti,ab,kw OR ctla4:ti,ab,kw OR pembrolizumab:ti,ab,kw OR nivolumab:ti,ab,kw OR atezolizumab:ti,ab,kw OR durvalumab:ti,ab,kw OR tremelimumab:ti,ab,kw OR ipilimumab:ti,ab,kw OR avelumab:ti,ab,kw OR cemiplimab:ti,ab,kw OR sintilimab:ti,ab,kw OR camrelizumab:ti,ab,kw OR tislelizumab:ti,ab,kw OR toripalimab:ti,ab,kw OR sugemalimab:ti,ab,kw OR penpulimab:ti,ab,kw OR serplulimab:ti,ab,kw OR envafolimab:ti,ab,kw OR zimberelimab:ti,ab,kw) AND ('first line':ti,ab,kw OR 'first-line':ti,ab,kw OR frontline:ti,ab,kw OR '1st line':ti,ab,kw OR '1l':ti,ab,kw OR 'treatment naive':ti,ab,kw OR 'treatment-naive':ti,ab,kw OR 'therapy naive':ti,ab,kw OR untreated:ti,ab,kw OR intervention*:ti,ab,kw OR 'previously untreated':ti,ab,kw OR 'systemic therapy':ti,ab,kw OR 'first therapy':ti,ab,kw) | |
| Web of science search strategy: | 410 articles |
| *#1* TS=(("hepatocellular carcinoma" OR HCC OR hepatoma OR "liver cancer" OR "primary liver cancer") NOT ("cholangiocarcinoma" OR "biliary tract cancer" OR "intrahepatic cholangiocarcinoma"))  *#2* TS=(advanced OR unresectable OR "non-resectable" OR inoperable OR metastatic OR "locally advanced" OR "intermediate stage" OR "BCLC B" OR "BCLC C" OR "stage III" OR "stage IV")  *#3* TS=("first line" OR "first-line" OR frontline OR "1st line" OR "1L" OR "treatment naive" OR "treatment-naive" OR "therapy naive" OR "previously untreated")  *#4* TS=("checkpoint inhibitor" OR "immune checkpoint inhibitor" OR "PD-1 inhibitor" OR "PD-L1 inhibitor" OR "CTLA-4 inhibitor" OR pembrolizumab OR nivolumab OR atezolizumab OR durvalumab OR tremelimumab OR ipilimumab OR sintilimab OR camrelizumab OR tislelizumab OR toripalimab)  *#5* TS=("phase 3" OR "phase III" OR "phase 3 trial" OR "phase III trial" OR  "randomized controlled trial" OR RCT OR randomized OR randomised OR  "randomly assigned" OR "clinical trial")  *#6 #1 AND #2 AND #3 AND #4 AND #5* | |
| Cochrane search strategy: | 867 trials |
| #1 MeSH descriptor: [Carcinoma, Hepatocellular] explode all trees  #2 MeSH descriptor: [Liver Neoplasms] explode all trees  #3 (hepatocellular near/3 (carcinoma or cancer or tumor* or tumour* or neoplasm* or malignan*)):ti,ab,kw  #4 ("liver cell" near/3 (carcinoma or cancer)):ti,ab,kw  #5 (HCC or hepatoma):ti,ab,kw  #6 #1 OR #2 OR #3 OR #4 OR #5  #7 MeSH descriptor: [Immune Checkpoint Inhibitors] explode all trees  #8 (checkpoint near/2 inhibitor*):ti,ab,kw  #9 ("immune checkpoint" near/2 (inhibitor* or blocker*)):ti,ab,kw  #10 ("PD-1" or "PD1" or "PD-L1" or "PDL1" or "CTLA-4" or "CTLA4"):ti,ab,kw  #11 (pembrolizumab or nivolumab or atezolizumab or durvalumab or tremelimumab or ipilimumab or avelumab or cemiplimab):ti,ab,kw  #12 (sintilimab or camrelizumab or tislelizumab or toripalimab or sugemalimab or penpulimab or serplulimab or envafolimab or zimberelimab):ti,ab,kw  #13 ("SHR-1210" or "IBI308" or "BGB-A317" or "JS001" or "CS1001" or "AK105" or "KN035" or "HLX10"):ti,ab,kw  #14 (sorafenib or lenvatinib or donafenib or regimen*):ti,ab,kw  #15 #7 OR #8 OR #9 OR #10 OR #11 OR #12 OR #13 OR #14  #16 (advanced or unresectable or "non-resectable" or inoperable or metastatic or "locally advanced" or "intermediate stage" or recurren*):ti,ab,kw  #17 ("BCLC" near/3 ("B" or "C" or "advanced")):ti,ab,kw  #18 ("Stage" near/3 ("III" or "IV" or "3" or "4" or "C")):ti,ab,kw  #19 #16 OR #17 OR #18  #20 ("first line" or "first-line" or "frontline" or "1st line" or "1L"):ti,ab,kw  #21 (first near/2 (line or therapy or treatment)):ti,ab,kw  #22 (previously near/3 (untreated or naive)):ti,ab,kw  #23 ("treatment naive" or "treatment-naive" or "therapy naive" or "untreated"):ti,ab,kw  #24 ("systemic" near/3 ("therapy" or "treatment" or "intervention")):ti,ab,kw  #25 #20 OR #21 OR #22 OR #23 OR #24  #26 MeSH descriptor: [Randomized Controlled Trial] explode all trees  #27 (randomized or randomised or randomly or placebo or "clinical trial" or trial or groups):ti,ab,kw  #28 #26 OR #27  #29 #6 AND #15 AND #19 AND #25 AND #28 | |

**Supplementary Table 2. Baseline characteristics of included trials**

| **Study**  **(Trial)** | **N**  **(randomized)** | **Region**  **(Asia/Global)** | **Median follow-up at OS analysis (months)** | **Experimental regimen** | **Control regimen** | **Male, n/N**  **(%)** | **ECOG 0, n/N**  **(%)** | **Child-Pugh A, n/N**  **(%)** | **BCLC C, n/N**  **(%)** | **MVI, n/N**  **(%)** | **EHS, n/N**  **(%)** | **HBV, n/N**  **(%)** | **HCV, n/N**  **(%)** | **AFP >=400 ng/mL, n/N (%)** | **Prior LRT, n/N**  **(%)** | **Double-blind** | **Strategy** |
| --- | --- | --- | --- | --- | --- | --- | --- | --- | --- | --- | --- | --- | --- | --- | --- | --- | --- |
| Llovet et al, 2023 (LEAP-002)^1^ | 794 | Global | 59 | Lenvatinib plus pembrolizumab | Lenvatinib | 644/794 (81%) | 538/794 (68%) | 790/794 (99%) | 612/794 (77%) | 133/794 (17%) | 492/794 (62%) | 385/794 (48%) | 181/794 (23%) | 251/794 (32%) | 397/794 (50%) | YES | PD-(L)1+TKI vs TKI |
| Zhou et al, 2025 (APOLLO) | 649 | Asia-dominant | 15 | Anlotinib plus penpulimab | Sorafenib | 551/649 (85%) | 369/649 (57%) | 600/649 (92%) | 527/649 (81%) | 266/649 (41%) | 404/649 (62%) | 546/649 (84%) | 23/649 (4%) | 319/649 (49%) | 360/649 (55%) | NO | PD-(L)1+TKI vs TKI |
| Yau et al, 2024 (COSMIC-312) | 649 | Global | 22 | Cabozantinib plus atezolizumab | Sorafenib | 546/649 (84%) | 419/649 (65%) | 649/649 (100%) | 437/649 (67%) | 197/649 (30%) | 355/649 (55%) | 191/649 (29%) | 203/649 (31%) | 228/649 (35%) | NA | NO | PD-(L)1+TKI vs TKI |
| Qin et al, 2025 (CARES-310) | 543 | Asia-dominant | 22 | Camrelizumab plus rivoceranib | Sorafenib | 457/543 (84%) | 236/543 (43%) | 543/543 (100%) | 465/543 (86%) | 92/543 (17%) | 355/543 (65%) | 405/543 (75%) | 51/543 (9%) | 196/543 (36%) | 311/543 (57%) | NO | PD-(L)1+TKI vs TKI |
| Yau et al, 2022 (CheckMate 459) | 743 | Global | 15 | Nivolumab | Sorafenib | 631/743 (85%) | 532/743 (72%) | 722/743 (97%) | 594/743 (80%) | 242/743 (33%) | 429/743 (58%) | 233/743 (31%) | 173/743 (23%) | 266/743 (36%) | 401/743 (54%) | NO | PD-(L)1 vs TKI |
| Zhao et al, 2025 (SCT-I10A-C301) | 346 | Asia-dominant | 20 | Finotonlimab plus SCT510 | Sorafenib | 301/346 (87%) | 158/346 (46%) | 322/346 (93%) | 277/346 (80%) | 136/346 (39%) | 211/346 (61%) | 308/346 (89%) | 17/346 (5%) | 164/346 (47%) | 112/346 (32%) | NO | PD-(L)1+VEGF(Ab) vs TKI |
| Ren et al, 2021 (ORIENT-32) | 571 | Asia-dominant | 10 | Sintilimab plus IBI305 | Sorafenib | 505/571 (88%) | 274/571 (48%) | 547/571 (96%) | 488/571 (85%) | 155/571 (27%) | 423/571 (74%) | 538/571 (94%) | 14/571 (2%) | 246/571 (43%) | 470/571 (82%) | NO | PD-(L)1+VEGF(Ab) vs TKI |
| Qin, 2023 (RATIONALE-301) | 674 | Asia-dominant | NA | Tislelizumab | Sorafenib | 570/674 (85%) | 364/674 (54%) | 672/674 (100%) | 524/674 (78%) | 100/674 (15%) | 417/674 (62%) | 409/674 (61%) | 85/674 (13%) | 251/674 (37%) | 515/674 (76%) | NO | PD-(L)1 vs TKI |
| Shi et al, 2025 (HEPATORCH) | 326 | Asia-dominant | 16 | Toripalimab plus bevacizumab | Sorafenib | 282/326 (87%) | 185/326 (57%) | 326/326 (100%) | 256/326 (79%) | 119/326 (37%) | 169/326 (52%) | 293/326 (90%) | 15/326 (5%) | 154/326 (47%) | 163/326 (50%) | NO | PD-(L)1+VEGF(Ab) vs TKI |
| Sangro et al, 2022 (HIMALAYA)^2^ | 1171 | Global | 62 | Tremelimumab plus Durvalumab/Durvalumab | Sorafenib | 987/1171 (84%) | 722/1171 (62%) | 1146/1171 (98%) | 948/1171 (81%) | 297/1171 (25%) | 624/1171 (53%) | 360/1171 (31%) | 321/1171 (27%) | 406/1171 (35%) | 117/1171 (10%) | NO | PD-(L)1+CTLA-4 vs TKI |
| Cheng et al, 2022 (IMbrave150)^3^ | 501 | Global | 16 | Atezolizumab plus bevacizumab | Sorafenib | 414/501 (83%) | 312/501 (62%) | 501/501 (100%) | 409/501 (82%) | 200/501 (40%) | 305/501 (61%) | 240/501 (48%) | 108/501 (22%) | 187/501 (37%) | 246/501 (49%) | NO | PD-(L)1+VEGF(Ab) vs TKI |
| Finn et al, 2025 (IMbrave152) | 669 | Global | 13 | Tiragolumab plus atezolizumab plus bevacizumab | Atezolizumab plus bevacizumab | 550/669 (82%) | 419/669 (63%) | 665/669 (99%) | 509/669 (76%) | NA | NA | 299/669 (45%) | 122/669 (18%) | 245/669 (37%) | 260/669 (39%) | YES | TIGIT+PD-(L)1+VEGF(Ab) vs PD-(L)1+VEGF(Ab) |
| Yau et al, 2025 (CheckMate 9DW) | 668 | Global | 35 | Nivolumab plus ipilimumab | Lenvatinib or sorafenib | 548/668 (82%) | 476/668 (71%) | 647/668 (97%) | 488/668 (73%) | 169/668 (25%) | 360/668 (54%) | 229/668 (34%) | 186/668 (28%) | 221/668 (33%) | 300/668 (45%) | NO | PD-(L)1+CTLA-4 vs TKI |
| Ren et al, 2025 (CS1003-305) | 534 | Asia-dominant | 43 | Nofazinlimab plus lenvatinib | Lenvatinib | NA | NA | NA | NA | NA | NA | NA | NA | NA | NA | YES | PD-(L)1+TKI vs TKI |
| Kudo et al, 2018 (REFLECT) | 954 | Global | 28 | Lenvatinib | Sorafenib | 806/954 (84%) | 605/954 (63%) | 946/954 (99%) | 758/954 (79%) | 199/954 (21%) | 586/954 (61%) | 479/954 (50%) | 217/954 (23%) | NA | NA | NO | TKI vs TKI |

Region is coded as Asia-dominant when the majority of participants were enrolled in Asian countries or the study population was predominantly Asian; otherwise coded as Global.

ECOG, Eastern Cooperative Oncology Group; BCLC, Barcelona Clinic Liver Cancer; MVI, macrovascular invasion; EHS, extrahepatic spread; AFP, alpha-fetoprotein; HCC, hepatocellular carcinoma; LRT, locoregional therapy; NA, not available.

1. LEAP-002 trial baseline characteristics and PFS/other efficacy outcomes were extracted from the 2023 primary publication; OS data were obtained from the ASCO 2025 long-term follow-up update.

2. HIMALAYA trial baseline characteristics and PFS/other efficacy outcomes were extracted from the 2022 primary publication; OS data were obtained from the 2025 five-year follow-up update.

3.IMbrave150 trial baseline characteristics were extracted from the 2020 primary publication; OS and PFS data are based on the updated data from 2022.

**Supplementary Table S3. Distribution of prespecified effect modifiers across strategy comparison classes**

| **Strategy comparison class** | **Trials (n)** | **HBV (%)** | **HCV (%)** | **MVI (%)** | **EHS (%)** | **Child–Pugh A (%)** | **ECOG 0 (%)** | **AFP ≥400 ng/mL (%)** | **Median OS follow-up (months)** |
| --- | --- | --- | --- | --- | --- | --- | --- | --- | --- |
| TKI vs TKI | 1 | 50 | 23 | 21 | 61 | 99 | 63 | NA | 28 |
| PD-(L)1 vs TKI | 2 | 46 (31–61) | 18 (13–23) | 24 (15–33) | 60 (58–62) | 98 (97–100) | 63 (54–72) | 36 (36–37) | 15 |
| PD-(L)1+CTLA-4 vs TKI | 2 | 32 (31–34) | 28 (27–28) | 25 (25–25) | 54 (53–54) | 98 (97–98) | 66 (62–71) | 34 (33–35) | 48 (35–62) |
| PD-(L)1+VEGF(Ab) vs TKI | 4 | 90 (79–91) | 5 (4–9) | 38 (34–39) | 61 (59–64) | 98 (95–100) | 52 (48–58) | 45 (42–47) | 16 (14–17) |
| PD-(L)1+TKI vs TKI | 5 | 62 (43–77) | 16 (8–25) | 24 (17–33) | 62 (60–63) | 100 (97–100) | 61 (54–66) | 36 (34–39) | 22 (22–43) |
| Overall | 14 | 50 (34–84) | 22 (5–23) | 27 (21–37) | 61 (55–62) | 99 (97–100) | 62 (54–65) | 36 (35–44) | 21 (15–33) |
| P value (Kruskal–Wallis) |  | 0.249 | 0.211 | 0.423 | 0.428 | 0.957 | 0.520 | 0.166 | 0.131 |

The number of contributing trials varied across variables because not all effect modifiers were consistently reported. Trials with missing values were excluded from the corresponding Kruskal–Wallis test.

**Supplementary Table S4. Model fit and heterogeneity assessments in the network meta-analysis and supplementary direct-evidence analysis**

Panel A. Model fit and between-study heterogeneity in the Bayesian network meta-analysis

| Analysis level | Endpoint | Model type | DIC | Between-study heterogeneity, τ |
| --- | --- | --- | --- | --- |
| Strategy-level | OS | Fixed-effect model | 19.71 | Fixed at 0; not estimated |
| Strategy-level | OS | Random-effects model | 21.06 | 0.05, 95% CrI 0.00–0.18 |
| Strategy-level | PFS | Fixed-effect model | 31.82 | Fixed at 0; not estimated |
| Strategy-level | PFS | Random-effects model | 27.23 | 0.12, 95% CrI 0.02–0.27 |
| Strategy-level | ORR | Fixed-effect model | 66.38 | Fixed at 0; not estimated |
| Strategy-level | ORR | Random-effects model | 55.15 | 0.41, 95% CrI 0.17–0.84 |
| Strategy-level | Grade ≥3 TRAEs | Fixed-effect model | 78.23 | Fixed at 0; not estimated |
| Strategy-level | Grade ≥3 TRAEs | Random-effects model | 56.45 | 0.34, 95% CrI 0.18–0.66 |
| Regimen-level | OS | Fixed-effect model | -10.18 | Fixed at 0; not estimated |
| Regimen-level | OS | Random-effects model | -10.42 | 0.34, 95% CrI 0.02–1.12 |
| Regimen-level | PFS | Fixed-effect model | -11.61 | Fixed at 0; not estimated |
| Regimen-level | PFS | Random-effects model | -11.44 | 0.34, 95% CrI 0.02–1.10 |

Panel B. Supplementary direct-evidence assessment of statistical heterogeneity across first-line regimen effects compared with sorafenib

| Outcome | I^2^ | P |
| --- | --- | --- |
| OS | 59% | 0.006 |
| PFS | 85% | ＜0.001 |

**Supplementary Table S5. DIC-based consistency assessment at the strategy level**

| Endpoint | Model | DIC consistency | DIC UME/inconsistency | ΔDIC consistency−UME |
| --- | --- | --- | --- | --- |
| OS | Random-effects | 21.06 | 21.05 | 0.01 |
| PFS | Random-effects | 27.23 | 27.25 | −0.02 |
| ORR | Random-effects | 55.15 | 55.11 | 0.04 |
| Grade ≥3 TRAEs | Random-effects | 56.45 | 56.61 | −0.16 |

**Supplementary Table S6. Posterior probability of each treatment strategy being ranked first at the strategy level**

| **Outcome** | **Treatment strategy** | **Pr(rank =1)** |
| --- | --- | --- |
| OS | PD-(L)1 | 0% |
| OS | PD-(L)1 + CTLA-4 | 6% |
| OS | PD-(L)1 + TKI | 2% |
| OS | PD-(L)1 + VEGF (Ab) | 92% |
| OS | TKI | 0% |
| PFS | PD-(L)1 | 0% |
| PFS | PD-(L)1 + CTLA-4 | 0% |
| PFS | PD-(L)1 + TKI | 9% |
| PFS | PD-(L)1 + VEGF (Ab) | 35% |
| PFS | TIGIT + PD-(L)1 + VEGF (Ab) | 56% |
| PFS | TKI | 0% |
| ORR | PD-(L)1 | 1% |
| ORR | PD-(L)1 + CTLA-4 | 10% |
| ORR | PD-(L)1 + TKI | 2% |
| ORR | PD-(L)1 + VEGF (Ab) | 25% |
| ORR | TIGIT + PD-(L)1 + VEGF (Ab) | 62% |
| ORR | TKI | 0% |
| Grade ≥3 TRAEs | PD-(L)1 | 100% |
| Grade ≥3 TRAEs | PD-(L)1 + CTLA-4 | 0% |
| Grade ≥3 TRAEs | PD-(L)1 + TKI | 0% |
| Grade ≥3 TRAEs | PD-(L)1 + VEGF (Ab) | 0% |
| Grade ≥3 TRAEs | TIGIT + PD-(L)1 + VEGF (Ab) | 0% |
| Grade ≥3 TRAEs | TKI | 0% |

**Supplementary Table S7. SUCRA values for OS and PFS in the regimen-level fixed-effect network meta-analysis**

| Regimen | OS SUCRA (%) | PFS SUCRA (%) |
| --- | --- | --- |
| Sint + IBI305 | 91 | 72 |
| Fino + SCT510 | 86 | 85 |
| Cam + Rivo | 81 | 77 |
| Atezo + Bev | 76 | 51 |
| Penpu + Anlo | 69 | 81 |
| Pem + Len | 59 | 77 |
| Nivo + Ipi | 59 | 60 |
| Tori + Bev | 53 | 45 |
| STRIDE | 53 | 22 |
| Nofa + Len | 45 | 92 |
| Nivolumab | 32 | 19 |
| Durvalumab | 32 | 9 |
| Tislelizumab | 32 | 3 |
| Lenvatinib | 17 | 50 |
| Atezo + Cabo | 12 | 38 |
| Sorafenib | 5 | 11 |
| Tira + Atezo + Bev | NA | 57 |

**Supplementary Table S8. Trial-defined grade ≥3 immune-related or immune-mediated adverse events in ICI-containing arms: descriptive summary by strategy**

| **Strategy** | **Trials reporting grade ≥3 irAEs (ICI arms)** | **Total N (ICI arms)** | **Total events (grade ≥3 irAEs)** | **Weighted incidence, 95% CI** | **Range (min)** | **Median** | **Range (max)** |
| --- | --- | --- | --- | --- | --- | --- | --- |
| PD-(L)1 + VEGF(Ab) | 2 | 392 | 38 | 9.7% (7.1–13.0%) | 6.2% | 9.2% | 12.2% |
| PD-(L)1 + TKI | 3 | 1099 | 108 | 9.8% (8.2–11.7%) | 6.5% | 8.9% | 16.5% |
| PD-(L)1 + CTLA-4 | 2 | 720 | 142 | 19.7% (17.0–22.8%) | 12.6% | 20.3% | 28.0% |
| PD-(L)1 monotherapy | 2 | 726 | 53 | 7.3% (5.6–9.4%) | 6.4% | 7.4% | 8.3% |

**Supplementary Table S9. Sensitivity analyses for the mixture-control weight in the CheckMate 9DW investigator’s-choice control arm**

| Outcome | Analysis | Assumption for w | HR for nivolumab plus ipilimumab versus sorafenib, median (95% CrI) |
| --- | --- | --- | --- |
| OS | Primary mixed-likelihood analysis | Fixed w=0.85 | 0.74 (0.58–0.93) |
| OS | Probabilistic sensitivity analysis | w∼Beta(276,51) | 0.74 (0.59–0.93) |
| OS | Non-informative stress test | w∼Beta(1,1) | 0.76 (0.61–0.94) |
| OS | Deterministic sensitivity scan | Fixed w=0.70 to 1.00 | 0.73–0.75 |
| PFS | Primary mixed-likelihood analysis | Fixed w=0.85 | 0.62 (0.50–0.78) |
| PFS | Probabilistic sensitivity analysis | w∼Beta(276,51) | 0.61 (0.49–0.78) |
| PFS | Non-informative stress test | w∼Beta(1,1) | 0.72 (0.51–0.96) |
| PFS | Deterministic sensitivity scan | Fixed w=0.70 to 1.00 | 0.57–0.66 |

**Supplementary Table S10. Sensitivity analysis after reclassifying camrelizumab plus rivoceranib as PD-(L)1 plus VEGF(Ab)**

| Endpoint | Treatment strategy | Effect measure | Effect estimate (95% CrI) |
| --- | --- | --- | --- |
| OS | PD-(L)1 + VEGF(Ab) | HR | 0.65 (0.57–0.73) |
| OS | PD-(L)1 + CTLA-4 | HR | 0.77 (0.67–0.89) |
| OS | PD-(L)1 + TKI | HR | 0.82 (0.73–0.92) |
| OS | PD-(L)1 monotherapy | HR | 0.85 (0.76–0.96) |
| PFS | TIGIT + PD-(L)1 + VEGF(Ab) | HR | 0.57 (0.41–0.80) |
| PFS | PD-(L)1 + VEGF(Ab) | HR | 0.58 (0.50–0.68) |
| PFS | PD-(L)1 + TKI | HR | 0.71 (0.60–0.82) |
| PFS | PD-(L)1 + CTLA-4 | HR | 0.89 (0.73–1.08) |
| PFS | PD-(L)1 monotherapy | HR | 1.01 (0.87–1.19) |
| ORR | PD-(L)1 + TKI | OR | 2.52 (1.70–4.11) |
| ORR | PD-(L)1 monotherapy | OR | 2.90 (1.79–4.69) |
| ORR | PD-(L)1 + CTLA-4 | OR | 3.86 (2.24–6.64) |
| ORR | PD-(L)1 + VEGF(Ab) | OR | 5.61 (3.71–8.79) |
| ORR | TIGIT + PD-(L)1 + VEGF(Ab) | OR | 6.82 (2.89–16.56) |
| Grade ≥3 TRAEs | PD-(L)1 monotherapy | OR | 0.27 (0.15–0.49) |
| Grade ≥3 TRAEs | PD-(L)1 + CTLA-4 | OR | 0.79 (0.38–1.61) |
| Grade ≥3 TRAEs | PD-(L)1 + VEGF(Ab) | OR | 1.22 (0.75–1.99) |
| Grade ≥3 TRAEs | PD-(L)1 + TKI | OR | 1.46 (0.85–2.51) |
| Grade ≥3 TRAEs | TIGIT + PD-(L)1 + VEGF(Ab) | OR | 1.67 (0.51–5.44) |

**Supplementary Table S11. Sensitivity analysis excluding IMbrave152**

Panel A. Strategy-level network meta-analysis results after exclusion of IMbrave152

| Endpoint | Treatment strategy | Effect measure | Effect estimate (95% CrI) |
| --- | --- | --- | --- |
| PFS | PD-(L)1 + VEGF(Ab) | HR | 0.60 (0.49–0.72) |
| PFS | PD-(L)1 + TKI | HR | 0.67 (0.57–0.78) |
| PFS | PD-(L)1 + CTLA-4 | HR | 0.89 (0.70–1.11) |
| PFS | PD-(L)1 monotherapy | HR | 1.01 (0.84–1.23) |
| ORR | PD-(L)1 monotherapy | OR | 2.90 (1.59–5.33) |
| ORR | PD-(L)1 + TKI | OR | 3.11 (1.97–5.25) |
| ORR | PD-(L)1 + CTLA-4 | OR | 3.89 (1.97–7.85) |
| ORR | PD-(L)1 + VEGF(Ab) | OR | 5.67 (3.22–10.63) |
| Grade ≥3 TRAEs | PD-(L)1 monotherapy | OR | 0.27 (0.17–0.43) |
| Grade ≥3 TRAEs | PD-(L)1 + CTLA-4 | OR | 0.78 (0.44–1.37) |
| Grade ≥3 TRAEs | PD-(L)1 + VEGF(Ab) | OR | 0.92 (0.59–1.43) |
| Grade ≥3 TRAEs | PD-(L)1 + TKI | OR | 1.73 (1.19–2.54) |

Panel B. Regimen-level network meta-analysis results after exclusion of IMbrave152

| Endpoint | Regimen | HR vs sorafenib (95% CrI) |
| --- | --- | --- |
| PFS | Penpu + Anlo | 0.52 (0.41–0.66) |
| PFS | Cam + Rivo | 0.54 (0.44–0.67) |
| PFS | Atezo + Cabo | 0.74 (0.56–0.98) |
| PFS | Lenvatinib | 0.65 (0.55–0.76) |
| PFS | Nofa + Len | 0.47 (0.36–0.61) |
| PFS | Nivolumab | 0.93 (0.79–1.10) |
| PFS | Tori + Bev | 0.69 (0.52–0.90) |
| PFS | Durvalumab | 1.02 (0.88–1.19) |
| PFS | STRIDE | 0.90 (0.77–1.05) |
| PFS | Atezo + Bev | 0.65 (0.53–0.81) |
| PFS | Pem + Len | 0.54 (0.43–0.68) |
| PFS | Sint + IBI305 | 0.56 (0.45–0.69) |
| PFS | Tislelizumab | 1.11 (0.92–1.33) |
| PFS | Fino + SCT510 | 0.50 (0.38–0.65) |
| PFS | Nivo + Ipi | 0.61 (0.49–0.77) |

**Supplementary Table S12. Strategy- and regimen-level network meta-analysis results after exclusion of the two conference abstracts**

Panel A. Strategy-level network meta-analysis results after exclusion of the two conference abstracts

| Endpoint | Treatment strategy | Effect measure | Effect estimate (95% CrI) |
| --- | --- | --- | --- |
| OS | PD-(L)1 + VEGF(Ab) | HR | 0.65 (0.55–0.76) |
| OS | PD-(L)1 + TKI | HR | 0.77 (0.67–0.88) |
| OS | PD-(L)1 + CTLA-4 | HR | 0.77 (0.65–0.92) |
| OS | PD-(L)1 monotherapy | HR | 0.85 (0.74–0.98) |
| PFS | PD-(L)1 + VEGF(Ab) | HR | 0.60 (0.49–0.73) |
| PFS | PD-(L)1 + TKI | HR | 0.65 (0.53–0.79) |
| PFS | PD-(L)1 + CTLA-4 | HR | 0.89 (0.69–1.13) |
| PFS | PD-(L)1 monotherapy | HR | 1.01 (0.82–1.24) |
| ORR | PD-(L)1 + VEGF(Ab) | OR | 5.70 (3.12–10.90) |
| ORR | PD-(L)1 + TKI | OR | 3.48 (1.99–6.46) |
| ORR | PD-(L)1 + CTLA-4 | OR | 3.89 (1.86–8.16) |
| ORR | PD-(L)1 monotherapy | OR | 2.90 (1.54–5.48) |
| Grade ≥3 TRAEs | PD-(L)1 + VEGF(Ab) | OR | 0.92 (0.58–1.46) |
| Grade ≥3 TRAEs | PD-(L)1 + TKI | OR | 1.84 (1.20–2.87) |
| Grade ≥3 TRAEs | PD-(L)1 + CTLA-4 | OR | 0.78 (0.43–1.41) |
| Grade ≥3 TRAEs | PD-(L)1 monotherapy | OR | 0.27 (0.16–0.44) |

Panel B. Regimen-level network meta-analysis results after exclusion of the two conference abstracts

| Endpoint | Regimen | HR vs sorafenib (95% CrI) |
| --- | --- | --- |
| OS | Penpu + Anlo | 0.69 (0.55–0.87) |
| OS | Cam + Rivo | 0.64 (0.52–0.79) |
| OS | Atezo + Cabo | 0.98 (0.78–1.24) |
| OS | Nivolumab | 0.85 (0.71–1.01) |
| OS | Tori + Bev | 0.76 (0.58–1.00) |
| OS | Durvalumab | 0.85 (0.73–1.00) |
| OS | STRIDE | 0.76 (0.65–0.89) |
| OS | Atezo + Bev | 0.66 (0.52–0.85) |
| OS | Lenvatinib | 0.92 (0.79–1.07) |
| OS | Pem + Len | 0.74 (0.59–0.91) |
| OS | Sint + IBI305 | 0.57 (0.43–0.75) |
| OS | Tislelizumab | 0.85 (0.71–1.02) |
| OS | Fino + SCT510 | 0.60 (0.44–0.82) |
| OS | Nivo + Ipi | 0.74 (0.58–0.93) |
| PFS | Penpu + Anlo | 0.52 (0.41–0.66) |
| PFS | Cam + Rivo | 0.54 (0.44–0.67) |
| PFS | Atezo + Cabo | 0.74 (0.57–0.98) |
| PFS | Nivolumab | 0.93 (0.79–1.10) |
| PFS | Tori + Bev | 0.69 (0.53–0.90) |
| PFS | Durvalumab | 1.02 (0.88–1.19) |
| PFS | STRIDE | 0.90 (0.77–1.05) |
| PFS | Atezo + Bev | 0.65 (0.53–0.80) |
| PFS | Lenvatinib | 0.65 (0.55–0.76) |
| PFS | Pem + Len | 0.54 (0.43–0.68) |
| PFS | Sint + IBI305 | 0.56 (0.45–0.69) |
| PFS | Tislelizumab | 1.11 (0.92–1.33) |
| PFS | Fino + SCT510 | 0.50 (0.38–0.65) |
| PFS | Nivo + Ipi | 0.61 (0.49–0.77) |

**Supplementary Table S13. Strategy-level fixed-effect network meta-analysis estimates and SUCRA rankings relative to TKI monotherapy**

Panel A. Strategy-level fixed-effect network meta-analysis estimates relative to TKI monotherapy

| Endpoint | Effect measure | Treatment strategy | Estimate (95% CrI) |
| --- | --- | --- | --- |
| OS | HR | PD-(L)1 + VEGF(Ab) | 0.65 (0.56–0.74) |
| OS | HR | PD-(L)1 + CTLA-4 | 0.77 (0.68–0.87) |
| OS | HR | PD-(L)1 + TKI | 0.78 (0.72–0.86) |
| OS | HR | PD-(L)1 monotherapy | 0.85 (0.77–0.94) |
| PFS | HR | TIGIT + PD-(L)1 + VEGF(Ab) | 0.58 (0.46–0.73) |
| PFS | HR | PD-(L)1 + VEGF(Ab) | 0.60 (0.53–0.67) |
| PFS | HR | PD-(L)1 + TKI | 0.68 (0.62–0.75) |
| PFS | HR | PD-(L)1 + CTLA-4 | 0.89 (0.79–1.00) |
| PFS | HR | PD-(L)1 monotherapy | 1.01 (0.92–1.11) |
| ORR | OR | TIGIT + PD-(L)1 + VEGF(Ab) | 6.40 (3.96–10.51) |
| ORR | OR | PD-(L)1 + VEGF(Ab) | 5.28 (3.75–7.59) |
| ORR | OR | PD-(L)1 + CTLA-4 | 3.79 (2.88–5.00) |
| ORR | OR | PD-(L)1 monotherapy | 2.89 (2.19–3.82) |
| ORR | OR | PD-(L)1 + TKI | 2.65 (2.14–3.29) |
| Grade ≥3 TRAEs | OR | PD-(L)1 monotherapy | 0.27 (0.22–0.33) |
| Grade ≥3 TRAEs | OR | PD-(L)1 + CTLA-4 | 0.78 (0.63–0.97) |
| Grade ≥3 TRAEs | OR | PD-(L)1 + VEGF(Ab) | 0.91 (0.74–1.12) |
| Grade ≥3 TRAEs | OR | TIGIT + PD-(L)1 + VEGF(Ab) | 1.25 (0.86–1.82) |
| Grade ≥3 TRAEs | OR | PD-(L)1 + TKI | 1.67 (1.44–1.93) |

Panel B. Strategy-level SUCRA rankings under the fixed-effect model

| Endpoint | Rank | Treatment strategy | SUCRA |
| --- | --- | --- | --- |
| OS | 1 | PD-(L)1 + VEGF(Ab) | 99% |
| OS | 2 | PD-(L)1 + CTLA-4 | 63% |
| OS | 3 | PD-(L)1 + TKI | 58% |
| OS | 4 | PD-(L)1 monotherapy | 30% |
| OS | 5 | TKI monotherapy | 0% |
| PFS | 1 | TIGIT + PD-(L)1 + VEGF(Ab) | 90% |
| PFS | 2 | PD-(L)1 + VEGF(Ab) | 88% |
| PFS | 3 | PD-(L)1 + TKI | 63% |
| PFS | 4 | PD-(L)1 + CTLA-4 | 39% |
| PFS | 5 | TKI monotherapy | 12% |
| PFS | 6 | PD-(L)1 monotherapy | 9% |
| ORR | 1 | TIGIT + PD-(L)1 + VEGF(Ab) | 97% |
| ORR | 2 | PD-(L)1 + VEGF(Ab) | 81% |
| ORR | 3 | PD-(L)1 + CTLA-4 | 61% |
| ORR | 4 | PD-(L)1 monotherapy | 35% |
| ORR | 5 | PD-(L)1 + TKI | 27% |
| ORR | 6 | TKI monotherapy | 0% |
| Grade ≥3 TRAEs | 1 | PD-(L)1 monotherapy | 100% |
| Grade ≥3 TRAEs | 2 | PD-(L)1 + CTLA-4 | 76% |
| Grade ≥3 TRAEs | 3 | PD-(L)1 + VEGF(Ab) | 59% |
| Grade ≥3 TRAEs | 4 | TKI monotherapy | 42% |
| Grade ≥3 TRAEs | 5 | TIGIT + PD-(L)1 + VEGF(Ab) | 22% |
| Grade ≥3 TRAEs | 6 | PD-(L)1 + TKI | 2% |

**Supplementary Table S14. SUCRA values for OS and PFS in the regimen-level random-effects NMA**

| Regimen | OS SUCRA (%) | PFS SUCRA (%) |
| --- | --- | --- |
| Sint + IBI305 | 73 | 64 |
| Fino + SCT510 | 70 | 72 |
| Cam + Rivo | 66 | 67 |
| Atezo + Bev | 63 | 53 |
| Penpu + Anlo | 60 | 69 |
| Pem + Len | 54 | 66 |
| Nivo + Ipi | 55 | 62 |
| Tori + Bev | 51 | 49 |
| STRIDE | 52 | 31 |
| Nofa + Len | 48 | 75 |
| Nivolumab | 41 | 29 |
| Durvalumab | 41 | 23 |
| Tislelizumab | 41 | 19 |
| Lenvatinib | 33 | 52 |
| Atezo + Cabo | 29 | 44 |
| Sorafenib | 23 | 21 |
| Tira + Atezo + Bev | NA | 55 |
